# Modulation of the response to immunotherapy in triple-negative breast cancer: the role of the microbiota and microbial metabolites in the tumor microenvironment

**DOI:** 10.1080/19490976.2026.2697600

**Published:** 2026-07-08

**Authors:** Lucía Serrano-García, Elisabeth Martínez-Salvador, Ana Belda-Marco, Clara Herrero-Oliva, Javier Cortés, Antonio Llombart-Cussac, Leonor Fernández-Murga

**Affiliations:** a Clinical and Molecular Oncology Laboratory, Hospital Arnau de Vilanova-Liria, FISABIO, Valencia, Spain; b IOB Madrid, Institute of Oncology, Hospital Beata María Ana, Madrid, Spain; c Translational Oncology Group, Facultad de Ciencias de la Salud, Universidad Cardenal-Herrera-CEU, Alfara del Patriarca, Spain

**Keywords:** Triple-negative breast cancer, microbiota, microbial metabolites, Immune checkpoint inhibitors, Immunotherapy resistance, Biomarkers

## Abstract

Triple-negative breast cancer is an aggressive and heterogeneous breast cancer subtype for which immune checkpoint inhibitors combined with chemotherapy have improved outcomes in selected patients. However, primary and acquired resistance remain common, underscoring the need to identify extrinsic, modifiable determinants of antitumor immunity. Increasing evidence indicates that the gut and tumor-associated microbiota shape systemic and intratumoral immune tone and influence the efficacy of cancer therapies. Beyond microbial composition, microbiota-derived metabolites—including short-chain fatty acids, indole–tryptophan derivatives, bile acids, polyamines, and other small molecules—can act as functional mediators linking microbial ecology to immune-cell programming and tumor biology. These metabolites modulate dendritic cell function, T-cell priming and fitness, myeloid polarization, inflammatory set points, and metabolic pathways within the tumor microenvironment, thereby potentially enhancing or constraining responses to chemoimmunotherapy. Importantly, while some studies propose intratumoral microbial effects, most clinically actionable evidence currently supports systemic gut-derived metabolites and immune tone modulation that secondarily shapes the TNBC tumor microenvironment. In this review, we synthesize current knowledge on (i) the immunobiology of triple-negative breast cancer (TNBC) relevant to microbiota-driven modulation, (ii) mammary and gut microbiome features reported in TNBC, and (iii) mechanistic pathways through which microbial metabolites may regulate antitumor immunity and immune checkpoint inhibitors (ICI) sensitivity. We also discuss methodological considerations for integrating microbiome profiling with metabolomics and immune phenotyping and evaluate emerging opportunities to leverage microbiota-derived metabolites as biomarkers and therapeutic targets. Finally, we highlight translational strategies—including diet, pre/probiotics, antibiotic stewardship, fecal microbiota transplantation, and metabolite-centric (“postbiotic”) approaches—and outline priorities for TNBC-focused, prospective multi-omics studies to move from associative signatures toward actionable interventions.

## Graphical abstract

Microbiota–metabolite–immune interactions shaping chemo-immunotherapy responses in triple-negative breast cancer (TNBC).

Gut and tumor-associated microbiota produce bioactive metabolites that modulate systemic and intratumoral immunity and therapeutic outcomes in breast cancer. The mammary microbiota produces metabolites and toxins, including steroid-related compounds, choline, *Escherichia coli*secretome, colibactin, and *Bacteroides fragilis* toxin (**BFT**). In parallel, the gut microbiota generates short-chain fatty acids (**SCFAs**), bile acids, indole/tryptophan derivatives, and bacterial toxins such as cytolethal distending toxin (**CDT**) and Shiga toxins. These microbial products modulate immune responses by promoting either antitumoral immunity (associated with eubiosis; increased M1 macrophages, balanced Th17/Treg responses, and enhanced CD8⁺ T cell activity) or protumoral immunity (associated with dysbiosis; increased M2 macrophages, N2 neutrophils, and Treg expansion). Additionally, microbial metabolites contribute to epigenetic regulation through mechanisms such as histone deacetylases (**HDAC**) inhibition, DNA methylation, and lactylation. These processes ultimately influence responses to chemo-immunotherapy, with microbial signatures serving as potential biomarkers distinguishing responders from non-responders. The figure also highlights key methodological considerations in microbiome research (e.g., low biomass and contamination risk) and potential microbiota-targeted therapeutic strategies, including probiotics, prebiotics, dietary interventions, fecal microbiota transplantation, and postbiotic approaches. [Created in Biorender: https://BioRender.com/rnllkcv].

**Figure:**
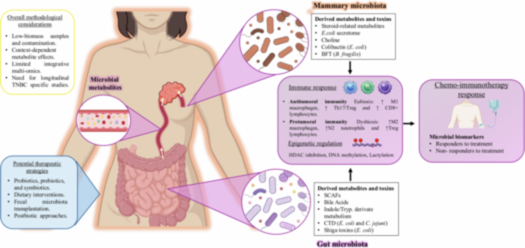

## 1. Introduction

Triple-negative breast cancer (TNBC) accounts for 15–20% of all breast cancers (BCs) and is among the most aggressive subtypes.^1^ Defined by the absence of estrogen and progesterone receptors (ERs and PRs) and low HER2 expression, TNBC lacks effective targeted therapies and is mainly treated with chemotherapy.^2^ The incorporation of immune checkpoint inhibitors (ICIs), alone or combined with chemotherapy, has improved outcomes in selected patients; nonetheless, a substantial proportion still develop primary or acquired resistance.^3^ This has prompted intense interest in additional, extrinsic determinants of therapeutic efficacy, particularly along the immune system–microbiome–metabolite axis.^4^

The TNBC tumor microenvironment (TME) is now viewed as a dynamic ecosystem in which tumor, stromal and immune cells interact to shape tumor progression and response to therapy.^5^ Within this context, the gut and breast microbiota have emerged as systemic regulators of cancer immunity. Microbial diversity and composition have been linked to antitumor immune priming, inflammatory tone, and sensitivity or resistance to ICIs in several tumor types, and these effects may be particularly relevant in immunogenic tumors such as TNBC.^6^ ^,^ ^7^

In contrast to recent reviews focused on breast cancer broadly/taxonomic descriptions, this review (i) centers on TNBC in the chemo-ICI setting, (ii) prioritizes metabolite-centric mechanisms, (iii) provides evidence stratification + clinical trial landscape in TNBC, and (iv) integrates the estrobolome/ERβ–GPER angle and our preliminary metabolomic observations.

Beyond compositional shifts, microbiota-derived metabolites—including short-chain fatty acids (SCFAs), indoles, polyamines, and bile acids—modulate immune cell function and tumor metabolism. These metabolites also influence signaling pathways involved in proliferation, apoptosis, and migration.^7^ Metabolomics allows comprehensive profiling of these molecules and their pathways, offering a rich source of candidate biomarkers and therapeutic targets.^8^ However, current evidence in TNBC remains fragmented. Most available data are extrapolated from other cancers and are limited by small cohorts, heterogeneous methodologies, and scarce integration of microbiome, metabolomic, and immune profiling. Notably, a recent pilot study has identified microbial and metabolic signatures associated with pathological complete response (pCR) and recurrence-free survival (RFS) in TNBC patients receiving chemoimmunotherapy.^9^

Our preliminary data are consistent with these observations, showing distinct microbial metabolomic profiles in TNBC patients according to their response to neoadjuvant chemoimmunotherapy.^10^ These findings support a functional link between the microbiota–metabolite axis and treatment outcomes, and underscore the need for a dedicated, TNBC-focused synthesis of the field.

In this review, we summarize key molecular and immunologic features of TNBC that make this subtype particularly sensitive to microbiota-mediated modulation. Then, we describe alterations in the mammary and gut microbiota in TNBC, followed by an in-depth analysis of how microbial metabolites influence innate and adaptive immunity, tumor metabolism and the TME. Next, we discuss therapeutic strategies aimed at targeting the microbiota and its metabolites—including probiotics, diet, antibiotics and fecal microbiota transplantation (FMT)—and examine emerging microbial and metabolomic biomarkers of prognosis and treatment response. Finally, we highlight methodological challenges and future translational directions, integrating recent clinical studies and our own preliminary findings in TNBC chemoimmunotherapy.

## 2. Methodology

This narrative literature review was conducted using PubMed, Google Scholar, and Web of Science to evaluate current evidence regarding the impact of the microbiome and its metabolites on the modulation of immunotherapy responses in female TNBC. The literature search included publications published between 1998 and 2026. The following keyword combinations were used: “triple negative breast cancer”, “breast cancer”, “breast microbiome”, “gut microbiome”, “microbiota”, “mammary microbiota”, “gut microbiota”, “immunotherapy”, “immune”, “inflammation”, “treatment”, “therapy”, “response to therapy”, “microbial metabolites”, “probiotics”, “dysbiosis”, “therapeutic strategies”, “metabolomics”, “immune response”, “immune checkpoint inhibitor”,“treatment response”, “epigenetic”, “microbial toxin” and “estrogen receptor”.

An initial screening of titles and abstracts was subsequently performed to identify studies aligned with the objectives of the review for further analysis and synthesis. Study selection primarily focused on the microbiota–metabolite–immune axis in TNBC. Nevertheless, due to the limited number of studies specifically addressing this topic, relevant studies on breast cancer more broadly were also considered when appropriate. Because low-biomass tumor microbiome studies are especially prone to technical bias, special emphasis was placed on contamination control, negative controls, sequencing methodologies, and reproducibility during literature evaluation.

## 3. TNBC features and therapeutic overview

### 3.1. Molecular profile and distinct subtypes

TNBC is defined by the absence of ERs and PRs and low HER2 expression and represents 15–20% of all BCs.^1^ ^,^ ^2^

Moreover, this disease is characterized by strong molecular, immune, and metabolic heterogeneity, at levels in which distinct subtypes have been described (Supplementary Table 1).^11-16^ Some of these subtypes have been associated with better prognosis and treatment response, including the molecular subtypes Basal-Like 1 (BL1),^13^ the basal-like immunoactivated (BLIA),^12^ and the metabolomic subtypes C1 and MPS2.^15^ ^,^ ^16^ Beyond these formal classifications, several studies have described recurrent functional metabolic states—such as glycolytic versus OXPHOS-high or lipid-driven phenotypes—that cut across transcriptomic and metabolomic subtypes.^11^ ^,^ ^16^ Together, these data highlight TNBC as a group of biologically distinct entities rather than a single disease and suggest that metabolic and immune features may interact with the microbiota and its metabolites to shape treatment outcomes.

### 3.2 Biological differences compared to other BCs

TNBC displays the highest tumor mutational burden (TMB) among BC subtypes, together with marked genomic instability, which promotes the generation of neoantigens potentially recognizable by the immune system^17^ or *TP53*, *BRCA1/2*, *MYC*, *RB1*, and PI3K/AKT/mTOR pathway mutations.^18^ ^,^ ^19^ These features confer sensitivity to DNA-damaging agents and PARP inhibitors. However, they also impose selective pressure that may promote immune escape.^20^ ^,^ ^21^

From an immunological perspective, TNBC is the most immunogenic BC subtype, with higher levels of tumor-infiltrating lymphocytes (TILs) and frequent tertiary lymphoid structures, both associated with improved prognosis and better responses to therapy.^22^ ^,^ ^23^ Nevertheless, the TME is often enriched in immunosuppressive cell populations (M2-like macrophages, N2 neutrophils, myeloid-derived suppressor cells, regulatory T cells, and exhausted CD8⁺ T cells) along with stromal components like cancer-associated fibroblasts (CAFs) that limit effective antitumor immunity.^24-27^

Multiple immune evasion pathways are active in TNBC, such as Notch and Wnt–β-catenin signaling, upregulation of immune checkpoints (PD-1/PD-L1, CTLA-4), and expression of immunosuppressive enzymes like IDO and arginase-1 (Arg-1). Importantly, these mechanisms may interact with microbial metabolites, providing a biological rationale for investigating how the microbiota–metabolite–immune axis influences immune responses and treatment outcomes in TNBC.^28-32^

### 3.3. Therapeutic overview of TNBC

Until recently, systemic treatment for TNBC relied almost exclusively on chemotherapy, surgery and radiotherapy with poor outcomes. The recognition of its relative immunogenicity and the presence of targetable DNA-repair defects have expanded therapeutic options.^1^ ^,^ ^17^ ^,^ ^19-21^ ^,^ ^33^ ^,^ ^34^

In the metastatic setting, clinical trials with chemoimmunotherapy IMpassion130 and KEYNOTE-355 demonstrated improved survival, particularly in patients with PD-L1–positive tumors.^35^ ^,^ ^36^ In early-stage disease, KEYNOTE-522 established neoadjuvant pembrolizumab plus chemotherapy as a standard of care, increasing pCR rates and overall survival (OS) compared with chemotherapy alone.^37^ Despite these advances, a substantial proportion of patients experience primary or acquired resistance to ICIs, highlighting the need for additional determinants of response.^3^ ^,^ ^35^ ^,^ ^37^ ^,^ ^38^

Novel approaches under investigation include antibody–drug conjugates (e.g. sacituzumab govitecan), PARP and PI3K/AKT inhibitors, antiangiogenic agents, and a range of immunotherapy strategies such as TILs-based therapies, chimeric antigen receptor (CAR)-T cells, oncolytic viruses and cancer vaccines.^10^

The microbiota and its metabolites have emerged as important modulators of treatment efficacy in TNBC, particularly in the context of chemoimmunotherapy. In a tumor setting characterized by high mutational burden, immunogenicity, and an immunosuppressive microenvironment, external regulators of immunity play a key role in shaping clinical outcomes.^39^ ^,^ ^40^ Both gut and tumor-associated microbiota can influence immune responses, affecting T-cell activation, myeloid cell function, and responses to chemotherapy and ICIs.^41^ ^,^ ^42^ Additionally, microbiota-derived metabolites can directly modulate immune and tumor cell signaling and metabolism, potentially reinforcing or counteracting mechanisms of immune escape and contributing to variability in treatment response.^43^

## 4. Characteristics of the microbiota in TNBC

Both mammary and gut microbial communities are altered in TNBC, with subtype-specific signatures that may influence tumor biology, antitumor immunity, and response to therapy.

### 4.1. The gut-mammary axis

The concept of a “gut-mammary axis” describes a two-way communication pathway linking gut microbiota and mammary tissue. This concept provides a framework to understand systemic effects, suggesting that BC is not merely a localized condition, but is also shaped by the gut environment, potentially involving the gut microbiome and the systemic circulation of microbial metabolites, immune cells and hormones.^44-46^

For example, toxins such as lipopolysaccharides (LPS) released by certain tumor-resident microorganisms, including *Staphylococcus* and *Streptococcus* could be transported to the gut, where they may disrupt intestinal barrier integrity. In parallel, gut dysbiosis may further disrupt intestinal homeostasis and function, promoting systemic inflammation and facilitating microbial dissemination to distant tissues, including the breast,^46^ ^,^ ^47^ potentially creating a self-reinforcing feedback loop. Other cancer-related detrimental effects caused by the gut microbiota include degradation of the tumor-suppressor gene *p53*, alteration of cell proliferation and survival pathways, activation of pro-inflammatory pathways, and dysregulation of the immune system.^48-50^

Therefore, the gut-mammary axis represents a mechanistic bridge between these two compartments, which may influence tumor initiation, progression, and therapeutic response. Although direct evidence in TNBC remains scarce, the gut-mammary axis provides a compelling framework for understanding how systemic microbial dynamics may shape the TME and therapeutic responses in this aggressive BC subtype, highlighting its potential as a target for microbiota-based interventions.

### 4.2. Mammary microbiome in TNBC

Breast tumors constitute low-biomass samples in which the microbial DNA burden approaches levels comparable to background contamination signals, rendering them highly susceptible to environmental and reagent-derived artifacts. Due to this limitation, it is critical to exercise caution when interpreting the microbiome associated with these samples in order to prevent erroneous inferences, as has been reported in certain instances.^51-53^

In healthy breast tissue, the predominant bacterial genera include *Staphylococcus*, *Corynebacterium*, *Propionibacterium*, *Streptococcus*, *Lactobacillus*, *Bacillus*, and *Micrococcus*.^54^ In the oncologic setting, this community becomes compositionally and functionally altered. Studies in BC cohorts suggests that the tumor microbiome (“oncobiome”) differs across BC subtypes, with a relative enrichment of Gram-negative species,^55^ while TNBC presents the lowest diversity among them.^56^ Furthermore, the oncobiome shows marked spatial and temporal heterogeneity. Distinct microbial distributions have been observed across benign, tumor-adjacent, and tumor tissues, and these patterns may also change during tumor progression.^57-60^ These alterations, collectively referred to as dysbiosis, may precede diagnosis, and potentially contribute to tumor progression. However, TNBC-specific evidence addressing these aspects remains limited. Most available studies in mammary and gut microbiota do not include subtype-stratified analyses, restricting our ability to assess their specific relevance in TNBC.

Emerging evidence further suggests that host factors such as race may influence the breast microbiome. In BC cohorts including TNBC, black women have been reported to exhibit a higher abundance of *Ralstonia*, a genus consistently associated with tumor tissue, and to experience worse TNBC-related clinical outcomes.^61^ In addition, an observational retrospective study reported differences in tumor-associated microbial composition between Black non-Hispanic (*n* = 7) and White non-Hispanic (*n* = 6) TNBC patients.^62^ White non-Hispanic patients showed higher microbial diversity in TNBC tissue compared with Black non-Hispanic patients. This effect was reversed in non-cancerous breast tissues. Distinct racial patterns in microbial composition were also observed: White women showed a higher abundance of *Phenylobacterium* in both normal and tumoral tissue, as well as increased abundance of *Lactobacillus* in tumor tissue. Conversely, Black women showed a greater abundance of an unclassified genus of *Plantococcaceae* in normal breast tissue. These differences in tumor-associated microbial composition between racial groups suggest that microbiota may contribute to BC disparities. Nevertheless, the small cohort sizes and limited TNBC-focused analyses currently available constrain the interpretation of these findings.

In TNBC, Nejman et al.^39^ reported decreased *Actinobacteria* and an increased prevalence of intracellular species such as *Achromobacter denitrificans*, *Bacillus_US21* and *Leptotrichia_US21*. In another cross-sectional observational study, Feng et al.^55^ described a predominance of *Actinobacteria* and *Pseudomonas*, while *Bacillus* and *Chlamydia* were detected in ~90% of TNBC samples and *Burkholderia* was associated with TNBC/basal-like tumors. Rashwan et al.^63^ presented a meta-analysis highlighting the enrichment in TNBC tissues with *Azospirillum oryzae* and two butyrate-producing bacteria, *Gemmiger formicilis* and *Anaerobutyricum soehngenii*. Certain genera appear to correlate with disease stage, including *Citrobacter* and *Bosea*, and *Fusobacterium nucleatum* has been linked to an immunosuppressive microenvironment and metastatic spread.^61^ ^,^ ^64^ ^,^ ^65^ By contrast, Banerjee et al.^66^ identified, in a retrospective observational study with computational multi-omics analysis, a multi-kingdom signature of bacteria, viruses, fungi and parasites associated with smaller tumors and longer progression-free survival (PFS) after treatment.

Consistent with these observations, tumor-associated microbial signatures have also been linked to treatment response in TNBC (Table 1). Pre-treatment biopsies showed that relative abundance of *Pandoraea pulmonicola* and *Brucella melitensis* appeared to be associated with complete response to neoadjuvant chemotherapy in a cohort of 88 samples, whereas *Geosporobacter ferrireducens*, *Streptococcus sanguinis*, *Nitrosospira briensis*, and *Plantactinospora* sp. BC1 were likely associated with distinct clinical outcomes, including residual disease.^67^ In addition, the presence of microbial DNA/RNA from taxa such as *Bacillus*, *Mucor*, *Nodaviridae*, *Toxocara*, and *Trichophyton* correlated with improved response to neoadjuvant chemotherapy and longer PFS over 200 d, highlighting the possible predictive potential of the tumor microbiota in TNBC.^66^

**Table 1.** Summary of prognostic microbial biomarkers of treatment response in triple-negative breast cancer (TNBC).

Microbial biomarkers	Evidence type	Sample (*n*)	Methodology	Treatment	Prognosis	Effect size metrics	Ref
**Pre-Treatment**							
*Akkermansia muciniphil*a; *Roseburia intestinalis; Enterocloster clostridioformi; Eubacterium eligens; Oscillibacter ruminantium*	Preclinical (murine model)	Fecal (*n* = 115)	Microbiome sequencing (16S + metagenomics) with functional validation in murine model (FMT)	Doxorubicin (CT)	Responders to treatment	N/A	[67]
*Alistipes putredinis; Enterorhabdus caecimuris*					Non-responders to treatment		
*Pandoraea pulmonicola; Brucella melitensis; Geosporobacter ferrireducens; Streptococcus sanguinis*	Translational (TNBC patients from data base)	Tumor (*n* from GEO = 88, *n* from TCGA = 115)	In silico tumor microbiome profiling from GEO/TCGA RNA-seq data (Kraken2), combined with immune deconvolution (CIBERSORTx), differential abundance analysis and machine learning–based predictive modeling (RF, SVM)	Standard NACT	pCR to treatment	Bacterial taxa enriched in pCR patients discriminated between pCR and residual disease with an AUC 0.67–0.98.	[68]
*Nitrosospira briensis; Plantactinospora* sp. BC1					Residual disease		
Alpha diversity	Translational (TNBC patients)	Fecal (*n* = 25)	Fecal microbiome profiling by 16S rRNA sequencing in TNBC patients undergoing neoadjuvant chemotherapy, with longitudinal sampling and diversity/compositional analysis	Paclitaxel + Cyclophosphamide	pCR to treatment	*Bacteroides eggerthii* enriched in non-pCR; 0% vs 1.4%.	[69]
*Bacteroides eggerthii*					Non-responders to treatment		
Alpha diversity; *Tannerellaceae; Enterohabdus; Bilophila*	Translational from Phase IIb ALICE Clinical trial cohort (NCT03164993)	Fecal (*n* = 70)	16S rRNA fecal microbiome sequencing in a randomized clinical trial (chemo-immunotherapy)	Atezolizumab (αPD-L1) + CT	Better progression free survival	Lower progression risk in high vs low alpha diversity; HR 0.34 vs 0.83.	[70]
*Bifidobacterium; Anaerostipes*					Non- responders to treatment		
*Bifidobacterium longum*	Translational from Phase II ARTEMIS Clinical trial cohort (NCT02276443)	Fecal (*n* = 85)	Fecal microbiome profiling by 16S rRNA sequencing (OTU-based, Mothur/SILVA), with alpha (Simpson index) and beta diversity (weighted UniFrac), and differential abundance analysis using DESeq2	Standard NACT	pCR to treatment	N/A	[71]
*Lachnospiraceae; Bacteroides thetaiotaomicron*					Residual disease		
*Bacteroides; Ruminnococcaceae*	Translational (TNBC patients)	Fecal (*n* = 30)	Fecal microbiome profiling by 16S rRNA sequencing in TNBC patients, with differential abundance analysis, and correlation with clinicopathologic features and treatment response	Standard NACT	pCR to treatment	N/A	[72]
*Bacteroides caccae*					Partial responders to treatment		
*Bacillus; Mucor; Nodaviridae; Toxocara; Trichophyton*	Translational (TNBC patients)	Tumor (*n* = 95-105)	Tumor microbiome profiling in FFPE breast cancer tissues using PathoChip microarray, with comparison across subtypes and integration with clinical and prognostic data	Non- specific	Responders to treatment	N/A	[66]
**Post-Treatment**							
INCREASED *Akkermansia muciniphila; Alistepes shahii; Prevotella copri; Oscillibacter* sp. 1–3*; Oscillospiraceae bacterium* VE202-24*; Bacteroides vulgatus; Enterorhabdus caecimuris*	Preclinical (murine model)	Fecal (*n* = 115)	Microbiome sequencing (16S + metagenomics) with functional validation in murine model (FMT)	Doxorubicin (CT)	Responders to treatment	N/A	[67]
REDUCED *Bacteroides uniformis; Lactobacillus johnsonii; Ruminococcus sp. 5_1_39BFAA*							

Bacterial signatures and associated metabolites detected in fecal or breast tumor tissue samples used to predict treatment response. Biomarkers are classified based on the timing of analysis: prior to treatment and post-treatment. **N/A**: not applicable, as no metrics were specified in the study. Abbreviations: **TNBC:** Triple Negative Breast Cancer; **CT**: chemotherapy; **NACT**: neoadjuvant chemotherapy; **pCR**: pathological complete response. **PD-L1**: Programmed Death-Ligand 1. **RD** = Residual disease; **PFS** = Progression free survival; **GEO** = Gene expression omnibus, **TCGA** = The Cancer Genome Atlas. **AUC**: Area under the curve; **HR**: Hazard ratio.

Considering the current methodological limitations associated with the analysis of low-biomass samples, collectively, these findings suggest that tumor-associated microbial signals may be linked to distinct immune and clinical phenotypes in TNBC, although causality remains uncertain and interpretation is limited by the low-biomass nature of these samples.

*Immunologic implications.* Although mechanistic data are still limited, several intratumoral taxa identified in TNBC have been associated with specific immune landscapes. *F. nucleatum* has been linked to myeloid-driven immunosuppression and reduced cytotoxic T-cell activity, whereas multi-kingdom signatures enriched in *Bacillus*, *Mucor*, *Nodaviridae* and *Trichophyton* correlate with earlier-stage disease, lower recurrence and improved PFS, consistent with a more inflamed, immunoreactive microenvironment.^61^ ^,^ ^64-66^ These observations support the concept that the breast oncobiome may fine-tune local immune tone—affecting TILs density, myeloid-cell polarization and checkpoint expression—and thereby influence sensitivity or resistance to immunotherapy in TNBC.

### 4.3 Gut microbiome in TNBC

The gut microbiota of healthy adults is primarily populated by anaerobic bacteria from the *Bacteroidetes* and *Firmicutes* phyla, which collectively account for 70–90% of the total microbial abundance.^68^ Other significant phyla include *Actinobacteria*, *Fusobacteria*, *Proteobacteria* and *Verrucomicrobia*.^69^ Numerous genera within these phyla contribute to gut homeostasis. For example, *Bifidobacterium* and *Lactobacillus* are commonly recognised as beneficial bacteria, as they support immune homeostasis, vitamin production and carbohydrate breakdown. Similarly, *Akkermansia muciniphila* promotes gut barrier integrity, preventing pathogen invasion in the gut, while *Faecalibacterium prausnitzii* is a key producer of butyrate, an anti-inflammatory SCFA that enhances gut barrier function.^68^ Disruptions in the composition and function of this balanced ecosystem, commonly referred to as intestinal dysbiosis, is increasingly recognized as a driver of tumor progression in preclinical TNBC models. Evidence indicates that gut microbial alterations can induce systemic immunosuppression, increase primary TMB, accelerate tumor growth and promote bone dissemination and metastatic spread in murine TNBC, supporting a direct link between dysbiosis and disease progression.^70-72^

Consistent with these findings, clinical studies have begun to identify distinct gut microbial signatures in TNBC patients. A case-control study reported increased *Anaerococcus* and decreased *Fischerella* and *Schizosaccharomyces* in TNBC patients relative to healthy controls.^73^ Terrisse et al.^40^ analyzed fecal samples from 76 early BC patients (24% TNBC) and observed that chemotherapy reshaped gut microbiota by enriching beneficial commensals such as *Dorea formicigenerans* while reducing the over-representation of “unfavorable” taxa, including *Clostridium asparagiforme, Bacteroides uniformis* and *Eggerthella lenta*, previously associated with BC diagnosis and poor prognosis. Similarly, Feng et al.^74^ reported *in silico* analysis higher levels of *Actinobacteria* and lower abundance of *Faecalibacterium*, a key anti-inflammatory genus, in TNBC patients compared with Luminal and HER2-positive subtypes.

Beyond descriptive associations, emerging evidence suggests that gut microbiota composition influences treatment response and clinical outcomes in TNBC (Table 1). In preclinical 4T1 mouse models, baseline enrichment of *A. muciniphila*, *Roseburia intestinalis* and other taxa correlated with better chemotherapy response, whereas *Alistipes putredinis* and *Enterorhabdus caecimuris* were enriched in non-responders. Post-treatment analyses further revealed that doxorubicin treatment exhibited enrichment of *A. muciniphila* and *E. caecimuris* whereas decreased probiotic *Bacteroides uniformis* and *Lactobacillus johnsonii*.^75^

In TNBC patients receiving neoadjuvant chemotherapy, higher baseline *α*-diversity and enrichment of specific taxa, including *Bacteroides eggerthii* and *Bifidobacterium longum* were associated with pCR, while *Bacteroides caccae* and *Gemellales* correlated with partial or absent responses.^76-78^ Similarly, in metastatic TNBC treated with atezolizumab plus chemotherapy, higher gut microbial diversity and enrichment of *Tannerellaceae*, *Bilophila*, and *Enterorhabdus* were linked to prolonged PFS and treatment response, while *Bifidobacterium* was correlated with poorer prognosis.^79^

Overall, these findings indicate that TNBC is associated with specific alterations in both gut and breast microbiota. However, most studies remain descriptive. Functional analyses are needed to clarify how these microbial communities and their metabolites contribute to tumor initiation, progression, and, crucially, to modulation of the immune response and therapeutic sensitivity in TNBC.

*Immunologic implications.* Gut microbiota alterations in TNBC are associated with systemic inflammation and immune modulation, influencing antitumor immunity. Dysbiosis characterized by loss of beneficial taxa and enrichment of pro-inflammatory or pathobiont bacteria may promote immunosuppressive cell populations and T-cell dysfunction. Preclinical evidence indicates that such microbial imbalances can impair responses to chemotherapy and ICIs, highlighting gut microbiota composition as an important regulator of immune contexture and treatment outcomes in TNBC.

### 4.4 Methodological and conceptual considerations

Characterizing mammary and gut microbiota in TNBC poses several technical and conceptual challenges.

As mentioned above, breast tumors constitute low-biomass samples. Consequently, methodological variables—including DNA extraction protocols, sequencing depth, and bioinformatic pipelines—can substantially influence the taxa detected.

Genera such as *Ralstonia*, *Sphingomonas* or *Pseudomonas* are frequently reported as common contaminants in low-biomass studies. As a result, these studies must primarily focus on contamination control to ensure the highest level of result accuracy and reliability. The methodological workflow should include prior identification of potential contamination sources; implementation of appropriate personal protective equipment; utilization of ultraclean, DNA-free reagents; application of rigorous decontamination protocols; incorporation of technical replicates and strict negative controls in parallel with each functional assay; integration of relative abundance approaches (e.g., metagenomics and 16S rRNA sequencing) with absolute quantification methods (e.g., qPCR or ddPCR) and complementary cell culture techniques; and the application of robust statistical analyses.^53^ ^,^ ^80-82^ Most human studies have relied on 16S rRNA amplicon sequencing in relatively small cohorts (typically ranging from 20 to 100 samples, although often less than 50), often with limited use of technical replicates, which complicates cross-study comparisons.^83-85^ In addition, many datasets are cross-sectional, making it difficult to infer causality or to distinguish whether microbiome alterations are drivers of tumor biology or consequences of cancer, prior therapies (antibiotics, chemotherapy, radiotherapy), diet or comorbidities. Geographic, dietary and host genetic differences further limit the generalizability of current findings.^86-88^

From an immuno-oncology perspective, there is also a scarcity of truly integrative analyses combining microbiome profiling with metabolomics and deep immune phenotyping.^89-91^ Only a few studies have correlated specific taxa with TILs, immune gene signatures, or response to immune checkpoint blockade in TNBC,^92^ and most available data focus on bacteria, while the contribution of the intratumoral mycobiome and virome remains largely unexplored.^93-95^ Longitudinal, multi-omics studies—including matched fecal, blood and tumor samples, high-dimensional immunophenotyping and functional validation in gnotobiotic or FMT models—will be essential to define mechanistic links between the microbiota, microbial metabolites and chemoimmunotherapy outcomes in TNBC.^41^ ^,^ ^43^ ^,^ ^91^ Such designs are particularly relevant to validate emerging microbial and metabolomic biomarkers and to place preliminary findings, including those from our group, in a robust translational framework.

## 5. Relevant microbial metabolites

Microbial communities in breast, tumor, and gut tissues can influence TNBC by releasing metabolites that alter tumor signaling and reshape antitumor immunity, affecting immune infiltration, inflammation, and response to ICIs (Table 2). However, because many of these pathways overlap with host and tumor metabolism, rigorous mechanistic and functional studies are needed to confirm microbial causality.

**Table 2.** Microbiota-derived (or microbiota-modulated) metabolites implicated in TNBC biology and potential links to immunotherapy .

Metabolite/axis	Compartment	Proposed microbial source	Main target/mechanism	Reported effect in TNBC	Link to ICIs	Evidence type	Sample (*n*)	Ref.
Pregnanetriol	TME	*Turicibacter*, *Clostridium* (processing)	Steroid-related metabolism	TNBC-associated metabolic signature (subtype-related)	Indirect (steroid–immune crosstalk plausible)	FFPE tissue metabolomics	FFPE samples (*n* = 22)	[96]
17β-estradiol-2,3-quinone (E2 quinone)	TME	*Turicibacter*, *Clostridium* (processing)	Steroid metabolites/DNA damage potential	TNBC-associated steroid-derived metabolite profile	Indirect	FFPE tissue metabolomics	FFPE samples (*n* = 22)	[96]
*E. coli* secretome (15 metabolites)	TME	*E. coli*	Tumor metabolic pathway rewiring	↑ tumor-cell survival	Potential resistance (not directly tested with ICI)	In vitro functional/metabolic	MDA-MB-231 (HTB-26) cells cultured (1 × 10^6^ cells/mL)	[97]
*Γ*-glutamyltryptophan *γ*-glutamylglutamate	TME	*Staphylococcus* (*S. aureus*)	CD8⁺ T-cell recruitment/activation	↑ CD8⁺ infiltration and activation	Potentially improves ICI responsiveness	Patient cohort and preclinical validation	Metabolome: breast cancer patients (*n* = 46) RNA sequencing: breast cancer patients (*n* = 360)	[92]
Choline → TMAO precursor	Systemic/TME	Microbial pathways (e.g., *Clostridium*)	PERK-dependent endoplasmic reticulum stress; pyroptosis	Pro-inflammatory cell-death signaling; improved ICI response reported	**Direct (associated with better ICI response)**	Clinical cohort	TNBC patients (*n* = 360)	[98]
Spliceostatin, terpenoids, pyochelin, riboflavin (mixture)	Tumor–microbe crosstalk	*P. aeruginosa* conditioned by TNBC media	Bioactive microbial compounds (antitumor potential)	Antitumor activity proposed	Indirect	Preclinical (conditioning experiments)	Breast cancer cell line (MDA-MB-231)	[99]
Lactate (lactic acid)	Gut/TME	*Lactobacillus* (attributed)	Immune evasion (acidification; effector T-cell suppression; myeloid skewing)	Promotes tumor progression via immune evasion	Potentially reduces ICI benefit	Association/mechanistic rationale (as described)	Murine cell line 4T1 and RNA-seq data from breast cancer patients (*n* = 255)	[100]
SCFAs (overall)	Gut/systemic	Fiber fermentation	Epigenetic/metabolic regulation	↓ viability and migration	Indirect (may support antitumor immunity)	Preclinical	Mixed human breast cancer cell lines	[101,102]
Butyrate	Gut/systemic	SCFA producers	HDAC inhibition/epigenetic effects	↓ lung metastases in 4T1; antiproliferative	Indirect	Preclinical (4T1)	Female Balb/CJ mice (*n* = 3-4/group)	[101,102]
Propionate (reduced)	Gut/systemic	↓ *F. prausnitzii* (producer)	“SCFA tone”	Lower SCFAs associated with increased cancer risk	Indirect	Patient association	Breast cancer patients (*n* = 44) and healthy subjects (*n* = 46)	[103]
Glucuronate/galacturonate (reduced)	Gut/systemic	Not specified	Microbial/host metabolism	Altered levels in TNBC patients	Indirect	Clinical metabolomics	Breast cancer patients (*n* = 14) and healthy subjects (*n* = 14)	[104]
Norvaline (reduced)	Gut/systemic	Not specified	Arg-1 inhibition; pro-apoptotic effects	↓ immunosuppression (via Arg-1) + apoptosis	Potentially improves ICI responsiveness	Clinical metabolomics + proposed mechanism	Breast cancer patients (*n* = 14) and healthy subjects (*n* = 14)	[104]
4-methylcatechol (increased)	Gut/systemic	*Rothia*, *Actinomyces*	Aromatic/phenolic metabolites	Elevated in TNBC murine models	Indirect	Preclinical	Murine cell line 4T1	[104]
Guaiacol (increased)	Gut/systemic	*Rothia*, *Actinomyces*	Aromatic/phenolic metabolites	Elevated in TNBC murine models	Indirect	Preclinical	Murine cell line 4T1	[104]
Indole-3-propionic acid (IPA)	Gut/systemic	*L. johnsonii*, *Clostridium*	Tcf7; ↑ cytotoxic T-cell activity	↑ apoptosis in TNBC organoids; ↑ immune receptor expression	**Direct (linked to improved ICI response)**	Preclinical + ICI association	Mixed murine models	[105,106]
Indoxyl sulfate (IS)	Gut/systemic	Indole pathway	iNOS induction; NRF2 inhibition; ↓ EMT	↓ EMT; oxidative/nitrosative stress; impaired energy metabolism	Indirect	Mechanistic (cell/BC; weaker in TNBC)	Mixed human breast cancer cell lines	[107]
Bile acid (BA) accumulation	Systemic	*Anaerococcus*, *Collimonas* (associated)	BA signaling/immunometabolism	Improved survival in BC datasets including TNBC	Indirect	Large patient dataset	Breast cancer patients (*n* > 1,000)	[108]
Lithocholic acid (LCA)	Systemic	Gut bacterial LCA producers	p53-mediated apoptosis; anti-angiogenesis; immunity-enhancing effects	↓ proliferation/angiogenesis; ↓ metastasis; LCA reduced in BC patients	Potentially improves ICI benefit	Preclinical + human associations	Mixed human breast cancer cell lines	[109,110]
Primary BA pathway ↑ in pCR vs AA pathway ↑ in non-responders	Systemic	Metabolic signature (microbiota-modulated)	Response stratification	Enriched BA metabolites in neo-ICI pCR	**Direct (pCR with ICI)**	Preliminary data (authors’ group)	TNBC patients (*n* = 28)	[10]
2,3-butanediol	Gut/systemic	Not specified	EMT inhibition; cytostatic effects	↓ EMT; cytostatic activity	Indirect	Preclinical	Murine cell line 4T1	[111]
Cadaverine	Gut/systemic	Bacterial lysine decarboxylation	↓ invasion/migration; ↓ CSC dedifferentiation	Reduced invasion, migration, CSC traits	Indirect	Preclinical + enzyme/survival correlation	Mixed human breast cancer cell lines	[112]
*β*-glucuronidase (estrobolome)	Gut/systemic	*Clostridia*, *Ruminococcaceae*, *Bacteroides*, *Lactobacillus*, *Escherichia/Shigella*	Estrogen deconjugation; enterohepatic recirculation	Hypothesized relevance in TNBC via ERβ/GPER	Indirect (potential immune intersection)	Mechanistic framework + microbial enzymology	ND	[113,114]
S-equol	Systemic	Bacterial metabolite (ERβ affinity)	ERβ-related signaling	~20% Ki-67 reduction in ~1/3 of cases	Indirect	Clinical study/trial in TNBC	TNBC patients (*n* = 39)	[115]
ERβ/GPER (clinical relevance)	TME	—	NFκB/RELA inhibition (ERβ); metastasis association (GPER)	High GPER: worse outcomes; ERβ: anticancer signaling	Indirect	Clinical cohort	Breast cancer patients (*n* = 360)	[116]

Abbreviations: **TME:** Tumor Microenvironment; **TNBC:** Triple Negative Breast Cancer, **ICI:** Immune Checkpoint Inhibitor; **TMAO:** Trimethylamine-*N*-Oxide; **SCFAs:** Short Chain Fatty Acids; **Arg-1**: Arginase-1; **Tcf7:** Transcription factor 7; **iNOS:** inducible Nitric Oxide Synthase; **NRF2:** Nuclear factor erythroid 2-related factor 2; **EMT:** Epithelial to Mesenchymal Transition; **BA:** Bile Acids; **BC:** Breast Cancer; **LCA**: Lithocholic acid; **pCR:** pathologic complete response; **AA:** Amino acid; **CSC:** Cancer Stem Cell; **ERβ**: Estrogen receptor β; **GPER:** G protein–coupled estrogen receptor, **FFPE**:Formalin-Fixed, Paraffin-Embedded; **MDA-MB**: MD Anderson Cancer Center- Mammary Breast; **SPF**: Specific Pathogen-Free:, **RNA-seq**: Ribonucleic acid-sequencing.

### 5.1 Intratumoral microbial metabolites

Although most research has focused on gut microbiome, emerging evidence shows that intratumoral bacterial metabolism in TNBC may be subtype-specific and functionally relevant. Mass spectrometry of formalin-fixed paraffin-embedded (FFPE) TNBC tissues revealed steroid-related metabolites (e.g., pregnanetriol, 17β-estradiol-2,3-quinone) which were associated with bacteria such as *Turicibacter* and *Clostridium*.^96^ AlMalki et al.^97^ identified *E. coli* secretome metabolites that could reprogram tumor energy metabolism and affect pathways associated with TNBC pathophysiology and tumor cell survival, while *Staphylococcus*-derived *γ*-glutamyl metabolites enhance CD8⁺ T-cell infiltration and activation after administration of *Staphylococcus aureus* in TNBC models.^92^ Similarly, Kadirareddy et al.^117^ reported that conjugated linoleic acid, a lipid derived from *Lactobacillus plantarum*, induces anti-proliferative and pro-apoptotic effects in the MDA-MB-231 cell line, a TNBC *in vitro* model via downregulation of the NF-κB pathway and overexpression of the Bax protein, highlighting its potential as a chemotherapeutic agent.

Also, clinical data support this axis: in a study with TNBC patients, Wang et al.^98^ reported that choline (trimethylamine *N*-oxide (TMAO) precursor, produced by *Clostridium*) correlated with PERK activation, pyroptosis, and improved ICIs responses, linking microbial metabolism to immune priming. Moreover, tumor–microbiota interactions appear bidirectional, as TNBC-conditioned media can induce bacteria like *Pseudomonas aeruginosa* to produce antitumor metabolites (e.g., spliceostatin, terpenoids, pyochelin, and riboflavin), reshaping the local metabolic landscape and increasing tumor metabolite production (e.g., Heme O, Ubiquinol-4 and citruline).^99^

*Methodological note.* Intratumoral microbiome research is prone to contamination and low-biomass artifacts, and FFPE-based analyses may not distinguish viable from residual microbial signals. Therefore, the most informative studies include strict negative controls alongside functional assays showing that identified metabolites modulate immune or tumor phenotypes.

**Figure 1.**
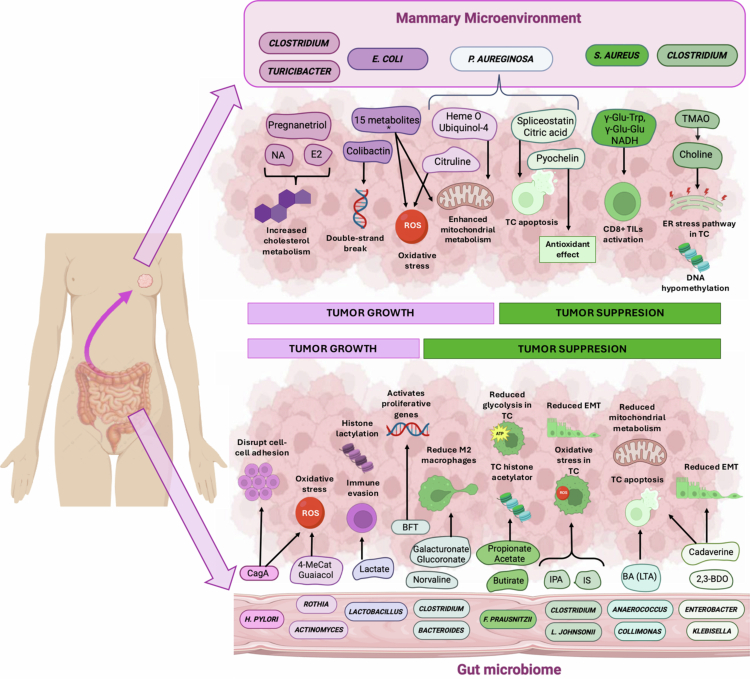
Effects of gut microbiota and mammary microenvironment–derived metabolites on triple-negative breast cancer (TNBC) progression. Gut microbiota and mammary microenvironment–associated bacteria produce a wide range of metabolites and toxins that can differentially modulate TNBC development through metabolic, redox, epigenetic, and immune-related pathways. Metabolites derived from mammary-associated bacteria, including *Clostridium*, *Escherichia coli*, *Pseudomonas aeruginosa* are predominantly associated with tumor-promoting effects (purple), in contrast, *P. aeruginosa and Staphylococcus aureus* are moreover associated with tumor-suppressive effects (green), through mechanisms such as enhanced cholesterol metabolism, oxidative stress, mitochondrial metabolism, immune improvement, and endoplasmic reticulum (ER) stress in tumor cells. In particular, a subset of biologically relevant metabolites derived of *E. coli* (*), including *N*-acetyl-L-methionine, methionine, nicotinamide riboside, nicotinamide adenine dinucleotide (NAD⁺), *N*-acetylneuraminic acid (sialic acid), mannose-1-phosphate, glutathionylspermidine, polyamines, dephospho-coenzyme A (dephospho-CoA), and bacterial lipids and phospholipids, contribute to oxidative stress and energy metabolism of tumor cells, that support tumor growth and survival. Conversely, metabolites produced by gut microbiota members such as *Clostridium*, *Bacteroides*, *Faecalibacterium prausnitzii*, *Lactobacillus johnsonii*, *Anaerococcus*, *Collimonas*, *Enterobacter*, and *Klebsiella*. These include fiber-derived short-chain fatty acids (acetate, propionate, and butyrate), glucuronate, galacturonate, L-norvaline, indole derivatives (indole-3-propionic acid and indoxyl sulfate), bile acids, cadaverine, and 2,3-butanediol. These metabolites contribute to reduced oxidative stress, inhibition of epithelial–mesenchymal transition (EMT), suppression of glycolysis and mitochondrial metabolism in tumor cells, induction of tumor cell apoptosis and epigenetic regulation and post-translational modification, decreased M2 macrophage polarization, and enhanced antitumor immune responses, including activation of CD8⁺ tumor-infiltrating lymphocytes (TILs). Conversely, 4-methylcatechol and Guaicol, mainly derived from *Actinomyces* and *Rothia*, contribute to producing reactive oxygen species (ROS) that promote tumor cell proliferation and survival. Overall, this schematic illustrates the dual and context-dependent role of microbial metabolites in shaping the TNBC microenvironment, highlighting the balance between tumor-promoting and tumor-suppressive microbial metabolic signals arising from distinct anatomical niches. Abbreviations: **E2**: 17beta-estradiol-2,3-quinone; **NA**: nutriacholic acid; ***γ*-Glu-Trp**: *γ*-glutamyltryptophan; ***γ*-Glu-Glu**: *γ*-glutamylglutamate; **NADH**: nicotinamide adenine dinucleotide; **TMAO**: Trimethylamine *N*-oxide; **4-MeCat**: 4-methylcatechol; **IPA:** indol-3-propionic acid; **IS**: indoxyl sulfate; **BA**: bile acid; **2,3-BDO**: 2,3-butanediol; **TIL**: tumor-infiltrating lymphocytes; **TC**: Tumor cell; **ER:** Endoplasmic reticulum; **ROS:** reactive oxygen species; **ATP**: Adenosine triphosphate; **EMT:** Epithelial–Mesenchymal Transition; **BFT**: *Bacteroides fragilis* toxin; **CagA**: Cytotoxin-associated gene A; ***P. aeruginosa**: Pseudomonas aeruginosa*; ***S. aureus***: *Staphylococcus aureus*; ***F. prausnitzii***: *Faecalibacterium prausnitzii*; ***L. johnsonii***: *Lactobacillus johnsonii.* [Created in Biorender: https://BioRender.com/dya7xss]

Current evidence indicates that intratumoral microbial metabolites can impact TNBC by both modifying tumor-cell metabolism and stress responses and by enhancing or suppressing CD8⁺ T-cell activity, affecting ICI efficacy (Figure 1).

### 5.2 Gut microbial metabolites

Beyond the TME, intestinal dysbiosis can influence TNBC through microbial metabolites released into the systemic circulation, which affect immune and metabolic signaling. For instance, in a preclinical murine model *Lactobacillus*-derived lactate is linked to tumor progression and immune evasion.^100^ Because lactate is also produced by tumor cells, distinguishing microbial vs tumor sources is essential; elevated levels in the TME can suppress effector T-cells, promote immunosuppressive myeloid programs, and potentially reduce ICIs effectiveness. Instead, several gut-derived metabolites can support antitumor immunity in TNBC. SCFAs from fiber fermentation reduce tumor viability and migration; in 4T1-bearing mice, butyrate decreased lung metastases and inhibited proliferation through epigenetic effects (histone deacetylases (HDAC) blockade).^101^ ^,^ ^102^ SCFAs-producing communities have been linked to better chemotherapy responses in TNBC mice models.^75^ Conversely, butyrate has been reported to have contradictory roles in cancer, with high concentrations being linked to apoptosis and tumor growth suppression, while low concentrations could stimulate cancer proliferation and migration. In TNBC, this phenomenon known as the butyrate-paradox could influence tumor aggressiveness and clinical outcomes.^63^ In a case-control observational study, reduced levels of SCFA-producing bacteria like *F. prausnitzii* correlate with lower SCFAs (especially propionate) and higher cancer risk.^103^ Zhu et al.^104^ also found decreased glucuronate, galacturonate, and norvaline (an Arg-1 inhibitor that promotes apoptosis); while metabolites such as 4-methylcatechol and guaiacol (associated with *Rothia* and *Actinomyces*) were elevated in TNBC models. Overall, gut metabolite profiles may influence tumor aggressiveness and immune pathways involved in ICI response. (Table 2).

The indole–tryptophan axis is a major immuno-oncology pathway linking microbial metabolism to T-cell function in TNBC. Microbes such as *L. johnsonii* and *Clostridium* spp. produce indole-3-propionic acid (IPA), which enhances cytotoxic T-cell activity via *Tcf7* and has been associated with improved ICIs response in multi-model and organoid studies.^105^ ^,^ ^106^ In TNBC organoids, IPA increased apoptosis and immune receptor expression.^105^ ^,^ ^106^ Consistently, metabolomic analyses identified tryptophan-derived metabolites as predictors of therapeutic outcome, with methylhistidine correlating with pCR, whereas microbial indole catabolites such as indole-3-acrylic acid (IAA) and indole-3-acetaldehyde (IAALD) were associated with unfavorable outcomes.^118^ Additionally, IPA and indoxyl sulfate induce oxidative and nitrosative stress and inhibit NRF2 signaling, reducing epithelial–mesenchymal transition (EMT) and tumor energy metabolism. Although clinically higher expression of indole-metabolite–producing enzymes correlated with improved overall BC survival, this association was less evident in TNBC,^107^ highlighting the need to clarify whether these effects are tumor-intrinsic, immune-mediated, or both.

Bile acids are clinically relevant immunometabolic mediators in TNBC. In a cohort of > 6,000 BC patients (including TNBC), higher bile acid accumulation (linked to *Anaerococcus* and *Collimonas*) was associated with better survival, whereas reduced bile acid metabolism (associated with *Lactobacillus, Ruegeria,* and *Marichromatium)* correlated with more aggressive disease.^108^ *In vitro* studies have shown that the gut bacteria-derived bile acid lithocholic acid (LCA) suppresses proliferation and angiogenesis, promotes p53-mediated apoptosis, strengthens antitumor immunity, and reduces metastasis. BC patients generally show reduced LCA activity.^109^ ^,^ ^110^ Consistently, our preliminary data suggest enrichment of primary bile acid metabolites in TNBC patients achieving pCR after neoadjuvant ICIs, whereas non-responders display increased phenylalanine–tyrosine metabolites.^10^

Other gut microbial metabolites can show antitumor effects: in 4T1 murine BC cell line, 2,3-butanediol inhibits EMT and exerted cytostatic effects;^111^ in several BC *in-vitro* cultures (specially MDA-MB-231) cadaverine reduces invasion, migration, and stemness. Reduced cadaverine-related enzymatic activity has been observed in patients, while higher lysine decarboxylase expression correlates with better survival.^112^ Generally speaking, microbiota-derived metabolites modulate proliferation, metastasis, and immunity in TNBC, highlighting their potential as a prognosis and treatment response biomarkers and therapeutic targets to improve ICIs responses (Figure 1).

### 5.3 Epigenetic modulation by microbial metabolites

Emerging evidence demonstrates that certain microbial metabolites induce epigenetic modifications implicated in TNBC. Epigenetic modifications are reversible biochemical alterations of chromatin or RNA that regulate gene expression without changing the DNA sequence, including DNA methylation, post-translational modifications, and non-coding RNA-mediated regulation.

The main metabolites associated with these epigenetic modifications are SCFAs, including butyrate, propionate, and acetate. Their primary mechanism involves HDAC inhibition, which may reactivate tumor suppressor genes silenced during tumorigenesis.^119-122^ Among SCFAs, butyrate is the most extensively studied and clinically relevant in oncology the context including TNBC, serving as a potent HDAC inhibitor (iHDAC). In breast cancer broadly, HDAC inhibition by SCFAs has been shown to derepress tumor suppressor and pro-apoptotic genes, suppress JAK/STAT-mediated inflammation, attenuate angiogenesis (VEGF/HIF), reduce EMT and metastasis through Wnt/β-catenin pathway downregulation via SFRP upregulation and promotes immune cell infiltration and cytokine production, although the underlying mechanisms remain incompletely characterized.^123-125^

Specific studies in TNBC have demonstrated that sodium butyrate (NaB) and sodium propionate (NaP) may inhibit the migratory and invasive capacity of MDA-MB-231 cells by altering the expression of EMT-associated genes and proteins through histone acetylation. These effects include the upregulation of E-cadherin and the downregulation of vimentin and *β*-catenin and the attenuation of MEK/ERK signaling.^126^ An *in vitro* study revealed that the combination of NaB with other HDAC inhibitors, such as suberoylanilide hydroxamic acid (SAHA), is capable of inhibiting TNBC cell proliferation by inducing cell cycle arrest at the G0/G1 phase and triggering mitochondrial apoptosis. This combination also suppressed the expression of mutant p53 protein without affecting the wild-type form through the inactivation of HDAC8, which leads to acetylation of residues 170–200 of the Yin Yang 1 (YY1) transcription factor, thereby preventing its binding to the mutant p53 (mtp53) promoter.^127^

Sharma et al.^128^ observed that the combination of NaB with the iHDAC sulforaphane (a cruciferous-derived compound) and genistein, a plant-derived product with DNA methyltransferase (DNMT) inhibitory activity, resulted in decreased proliferation with cell cycle arrest at the G2/M phase and increased necrosis and apoptosis in MDA-MB-231 cells. The synergistic mechanism underlying these effects was mediated by methylation of histone H3 at lysine 9 (H3K9me), methylation of histone H3 at lysine 27 (H3K27me), and through the induction of histone acetyltransferase activity.^129^ Figueiredo-Rodrigues and colleagues^130^ observed that both NaB and another iHDAC, trichostatin A (TSA), induced an upregulation of pyruvate kinase in an *in vitro* TNBC cell model, suggesting a tumor metabolic reprogramming. Additionally, both agents reduced anchorage-independent growth, suggesting reduced proliferation of cells with invasive potential. Among microbial metabolites with antitumoral capacity, TMAO has also been identified as an epigenetic modulator. TMAO acts through the disruption of the methionine cycle and the dynamic remodeling of chromatin states via histone methylation and acetylation.

Furthermore, TMAO functions as a non-competitive inhibitor of adenosylhomocysteinase (S-adenosyl-L-homocysteine hydrolase), leading to the accumulation of S-adenosylhomocysteine (SAH) and a consequent reduction in DNA methylation capacity.^131^ ^,^ ^132^ However, evidence regarding the effects of this epigenetic mechanism specifically in the context of TNBC remains lacking. Therefore, further investigation is warranted to determine whether processes such as the stimulation of antitumor immunity attributed to TMAO (see Section 5.1) may have an underlying epigenetic mechanism.

On the other hand, certain microbial metabolites such as lactate exert epigenetic effects with protumoral consequences in TNBC models (*in vitro* and *in vivo*). Lactate induces histone lactylation, a post-translational modification that increases chromatin accessibility and promotes the transcription of otherwise silenced oncogenes. Lactylated histones, including H3K9la, H3K14la, and H3K18la, have been associated with various malignant features, including immune modulation—as certain cellular states are dependent on histone lactylation—and metabolic reprogramming.^133^ Notably, lactate in the tumor microenvironment may originate from both tumor cell glycolysis (via the Warburg effect) and bacterial fermentative metabolism (e.g., *Lactobacillus* species). This dual origin complicates mechanistic interpretation because the relative contribution of host- versus microbiota-derived lactate remains unclear. These findings should therefore be interpreted with caution, and future studies employing isotope tracing or germ-free models are needed to dissect the metabolic source driving protumoral histone lactylation in TNBC.

The toxin produced by enterotoxigenic *Bacteroides fragilis* (BFT), a gut bacteria present in malignant mammary tissue, constitutes another microbial metabolite involved in the 56 differentially methylated CpG, 26 hypomethylated genes and hypermethylation of 156 genes, five of them were tumor suppressor genes—NF2, RSK3, FAT4, DCN, and DOK2—. Notably, DNMT inhibitors (azacitidine) and iHDAC TSA were able to rescue the expression of these five genes, resulting in a reduction of tumor migration and invasion in MDA-MB-231 cells treated with BFT.^134^

Additional microbial metabolites with epigenetic modulatory capacity have been described, including polyamines, acetate, polyphenols, vitamin K, and B-group vitamins. Although these compounds have been shown to participate in processes such as methylation, acetylation–deacetylation, biotinylation, phosphorylation, and non-coding RNA regulation, no specific studies addressing their involvement in TNBC currently exist, underscoring the need for further research in this area.^135^ Collectively, these findings highlight the therapeutic potential of microbial metabolites at the epigenomic level. Accordingly, combinatorial strategies integrating microbiota modulation with epigenetic-targeted therapies may open the door to novel and as yet underexplored therapeutic approaches.^136^

### 5.4 Role of microbial toxins

Among the diverse mechanisms involved, the intestinal microbiota can also produce a variety of toxins that modulate cancer-related processes. These microbial toxins may exert both pro-tumorigenic and anti-tumorigenic effects, thereby playing a central role in shaping tumor development and progression. Understanding this duality is essential to grasp the complexity of host–microbiome interactions and for the development of microbiome-based therapeutic strategies.

As demonstrated in several preclinical mechanistic studies (*in vitro* and *in vivo*), genotoxins (toxins capable of damaging DNA) such as colibactin, produced by certain strains of *E. coli*, as well as some staphylococcal species associated with breast tissue, induce DNA double-strand breaks, resulting in genomic instability and the progressive accumulation of mutations, facilitating tumor development.^48^ ^,^ ^68^ ^,^ ^137-139^ Similarly, the cytolethal distending toxin (CDT), produced by bacteria such as *E. coli* and *Campylobacter jejuni*, leads to transient disruption of cell cycle progression and DNA integrity, thereby promoting mutagenesis and contributing to tumorigenesis.^48^ These DNA double-strand breaks compromise genomic integrity, a key driver of carcinogenesis.

Conversely, other microbial toxins may modulate signaling pathways involving cell proliferation and survival. Some examples include CagA (Cytotoxin-associated gene A) from *H. pylori*, FadA (Fusobacterium adhesin A) from *F. nucleatum*, and metalloproteinases (MPs) from *B. fragilis*, which disrupt cell–cell adhesion and activate *β*-catenin signaling, thereby facilitating carcinogenic transformation.^48^ ^,^ ^140^ As discussed in the epigenetic section on epigenetic modulation, the enterotoxigenic *B. fragilis* toxin (BFT) may promote tumor progression and contribute to poor clinical outcomes through the epigenetic silencing of tumor suppressor genes (see section 5.3).^68^ ^,^ ^137^ ^,^ ^138^ ^,^ ^140^

In contrast, certain bacterial toxins exhibit tumor-selective cytotoxicity, particularly in TNBC cells, and may exert anti-tumorigenic and therapeutic effects. For instance, LT-IIc, a type II heat-labile enterotoxin produced by *E. coli,* displays tumor-selective cytotoxicity in TNBC cell lines (e.g., MDA-MB-231) while sparing non-malignant cells. Through its dual mechanism in tumor cells of autophagy induction and inhibition of lysosomal degradation, LT-IIc promotes the accumulation of autophagolysosomes, leading to activation of cellular stress pathways and the induction of apoptosis, necrosis, and necroptosis.^141^

Other toxins, such as Shiga toxins (Stx1 and Stx2), produced by *E. coli*, exert their effects by inhibiting ribosomal function and suppressing protein synthesis, once they are selectively internalized into TNBC cell lines through Gb3 membrane receptor. Therefore, they may induce cellular stress responses, apoptosis, and autophagy, while reducing cell proliferation, migration, and viability.^142^

Regarding toxins with anticarcinogenic potential, enterotoxins derived from *Clostridium perfringens* can target membrane-associated proteins such as claudins, which are frequently overexpressed in tumor cells, inducing pore formation and selective cytotoxicity, whereas cytolysin-producing bacterial strains exert membrane-disruptive activity that ultimately leads to tumor cell death. Although these effects have been primarily demonstrated in experimental models, they highlight the therapeutic potential of microbial toxins in cancer treatment.^140^ ^,^ ^143^

Microbial toxins not only influence tumor biology but also modulate therapeutic responses. For example, preclinical studies have demonstrated that nanotechnology-based approaches can therapeutically exploit microbiota-associated molecules, including microbial products such as LPS, to reshape the tumor–immune microenvironment. Despite its established association with immune suppression and liver metastasis, LPS can be strategically engineered or delivered to enhance anti-tumor immune responses and increase the effectiveness of PD-L1 checkpoint inhibition. These findings highlight that microbial toxins are not only involved in tumor progression but may also serve as valuable tools in cancer therapy.^140^

Overall, these findings highlight the dual and context-dependent role of microbial toxins in TNBC, acting either as drivers of tumor initiation and progression or as potential therapeutic agents capable of selectively targeting tumor cells and modulating anti-tumor immune responses. Although most evidence remains preclinical, the growing understanding of toxin-mediated host–tumor–microbiome interactions may open new avenues for biomarker discovery and microbiome-based therapeutic strategies in TNBC.

### 5.5 Influence of gut microbiota on estrogen regulation

The gut microbiota regulates systemic estrogen availability through the estrobolome, a collection of microbial enzymes—particularly *β*-glucuronidase and sulfatases—that deconjugate estrogens and promote their enterohepatic recirculation. Following hepatic conjugation and biliary excretion, conjugated estrogens reach the intestinal lumen, where bacterial enzymatic activity restores them to their active forms, enabling their reabsorption into the circulation and subsequent distribution to peripheral tissues, including the TME.^113^

Within the TME, these reactivated estrogens can engage non-classical estrogen signaling pathways. Although TNBC, as well as ER-low tumors (1–10%), lack ERα/PR/HER2 expression, alternative estrogen receptors such as estrogen receptor beta (ERβ) and the G protein–coupled estrogen receptor (GPER) may still influence tumor biology and immune responses.^144^ Clinically, a cohort of 360 TNBC patients shows high GPER expression that has been associated with increased metastasis and poor survival, whereas ERβ activation has been linked to tumor-suppressive effects.^116^

Mechanistically, estrogen-derived activation of ERβ and GPER triggers downstream signaling pathways, including MAPK/ERK, PI3K/AKT, and NF-κB. ERβ is primarily activated by microbiota-influenced estrogenic metabolites, such as 17β-estradiol (E2), 2-hydroxyestradiol (2-OHE2), and estriol (E3), and is predominantly expressed in normal breast tissue where it is often downregulated during tumor progression.^145^ ^,^ ^146^ However, in TNBC, ERβ has been shown to suppress NF-κB signaling through epigenetic reprogramming of EZH2, promoting repression of NF-κB target genes, thereby reducing pro-inflammatory and pro-survival transcriptional programs and limiting tumor progression while reshaping the TME toward a less pro-tumorigenic state.^147^ Clinically, S-equol—an ERβ-affine bacterial metabolite—reduced Ki-67 by ~20% in one-third of TNBC patients.^115^

In contrast, GPER can be activated by estrogenic ligands such as E2, estrone (E1), E3, and 2-OHE2.^148^ GPER activation has been associated with tumor-promoting effects, including epidermal growth factor (EGFR) transactivation and subsequent activation of MAPK/ERK and PI3K/AKT signaling pathways, leading to enhanced proliferation, migration, and metastatic potential. Furthermore, GPER signaling contributes to the modulation of the tumor immune microenvironment by regulating cytokine production, immune cell recruitment, and inflammatory responses, ultimately correlating with worse clinical outcomes.^149^

In this context, microbiota-driven estrogen metabolism may contribute to shaping the TME even in hormone receptor–negative tumors such as TNBC; nonetheless, the number of studies directly supporting this relationship remains limited. TNBC tumors also display elevated steroid-derived compounds (such as nutriacholic acid and oxidized estradiol) capable of inducing DNA damage.^96^ Although these direct causal links are limited, TNBC-associated bacteria (e.g., *Clostridia*, *Ruminococcaceae, Bacteroides, Lactobacillus, Escherichia/Shigella*) encode *β*-glucuronidase and may affect estrogen recirculation.^114^

Although the role of the estrobolome in regulating estrogen metabolism is comparatively well characterized, direct evidence linking microbiota-driven estrogen modulation to TNBC remains scarce, with current understanding largely extrapolated from broader breast cancer studies and indirect mechanistic data.

### 5.6 Methodological considerations in gut microbial metabolite research in TNBC

Despite growing evidence that gut microbial metabolites influence TNBC biology and immunotherapy responses (Table 2), methodological limitations still hinder clinical translation. Recognizing these challenges is essential for interpreting current data and guiding future studies.

A key challenge in metabolite research is distinguishing microbial-derived metabolites from host-produced ones, particularly for molecules that originate from both tumor cells and bacteria, like lactate, as noted in section 5.2. ^150^ ^,^ ^151^ This dual origin complicates attribution of biological effects and mechanistic interpretation.

Advanced techniques like imaging mass spectrometry, and spatial multi-omics may help map metabolites to immune and tissue contexts,^151^ but they are resource-intensive and not widely used in TNBC studies.

Microbial metabolites are often context-dependent, influenced by receptor interactions, dose, tissue niche, and tumor type.^150-152^ Bidirectional effects can be either tumor-suppressive or protumorigenic depending on the setting. For instance, SCFAs -butyrate can inhibit HDAC enzymes and induce apoptosis, conferring multidrug resistance-^152-154^ and secondary bile acids, such as deoxycholic acid (DCA), which is generally protumorigenic, versus LCA, which exhibits anticancer properties.^150^ These paradoxical effects highlight the need for personalized strategies considering individual microbiome profiles, metabolite context, host-microbiome interactions and tumor characteristics.^152^

Mechanistic insights into gut microbial metabolites are largely derived from other cancers, while evidence in BC (including TNBC) remains limited.^151^ Most evidence on SCFAs, bile acids, and indole metabolites comes from other tumor types or unstratified BC cohorts. Associations between indole-pathway enzymes and survival are weaker in TNBC, and links between TNBC microbiota and estrogen metabolism remain limited (section 5.5). Future work must use TNBC-focused models (e.g. organoids, mouse models) and stratified clinical cohorts to define relevant metabolite–immune interactions.

Measuring metabolites in stool, blood, and tumor tissue is challenging,^151^ ^,^ ^155^ ^,^ ^156^ as fecal levels may not reflect systemic or intratumoral exposure, and metabolite stability varies with sample handling.^150^ ^,^ ^155^

Defining exposure–response effects require advanced functional platforms that control confounding variables, such as immune-competent organoid co-culture systems.^150^ In the estrobolome, fecal *β*-glucuronidase activity varies widely between healthy and cancer patients^157-159^ and alone cannot reliably predict estrogen reactivation or cancer risk without additional clinical and metabolic biomarkers.^159^ ^,^ ^160^

Although gut microbial metabolites are promising ICIs biomarkers (section 5.2), validation is challenging.^151^ ^,^ ^155^ Small single-center studies often lack generalizability due to diet, geography, antibiotics exposure, and host-microbiome variability.^161^ Estrogen metabolism and *β*-glucuronidase activity are highly variable, apparently depending on specific strains, diet, and host genetics.^160^ ^,^ ^162^ ^,^ ^163^ Reliable clinical application will require large, multi-center cohorts with standardized protocols and rigorous analysis. Developing targeted interventions also depends on identifying which metabolite–receptor pathways to prioritize.^151^

Future studies should combine single-cell and spatial multi-omics with 16S sequencing, metagenomics, and metabolomics, using longitudinal sampling to track therapy-related changes. Advanced computational tools like network analysis and machine learning can help identify actionable metabolites–immune signatures,^151^ ^,^ ^155^ while standardized protocols improve reproducibility. Mechanistic validation in gnotobiotic models, organoids, and patient-derived xenografts is essential to move from correlation to causation and enable precision microbiome-based strategies in TNBC.

Current evidence on microbiota-derived metabolites in TNBC reveals a clear gradient of translational readiness. Among them, SCFAs currently represent the most clinically mature and translationally promising group, supported by relatively consistent evidence regarding their immunomodulatory, epigenetic, and tumor regulatory effects. Additionally, clinically supported observations include choline/TMAO- related pathways associated with immune activation and improved ICI responses, bile acid metabolism correlate with patient survival, and S-equol with antiproliferative effects.

In contrast, most other metabolites remain at a preclinical or exploratory stage. These include intratumoral *γ*-glutamyl peptides, indoles, cadaverine, 2,3 butanediol, lithocholic acid, NaB/NaP, and several microbial toxins, which have shown effects on CD8+ T cell activity, immune modulation, epigenetic regulation, EMT-related pathways, or tumor-selective cytotoxicity in experimental models.

Together, these findings highlight that while several microbial metabolites are emerging as promising candidates in TNBC, most evidence remains preclinical and requires further mechanistic and clinical validation before therapeutic translation.

## 6. Mechanisms of intestinal microbiota interaction with TNBC

### 6.1 Modulation of the immune response by the gut microbiota in TNBC

The gut microbiota interacts with innate and adaptive immunity, influencing immune cell differentiation, cytokine production, and overall immune homeostasis. It contributes to intestinal mucosa integrity and gut-associated lymphoid tissue (GALT) maturation.^164^ Microbial-associated molecular patterns (MAMPs) such as LPS, SCFAs, and peptidoglycans are recognized by pattern recognition receptors (PRRs), including Toll-like receptors (TLRs) and Nucleotide-binding Oligomerization Domain-like Receptors (NLRs), on antigen-presenting cells (APCs).^49^ Immune mediators originating in the gut may reach the TME, where they shape the biology of tumor cells and responses to immunotherapies.^165^ As noted above, TNBC is typically characterized by a pro-metastatic and immunosuppressive TME, correlating with poor response to ICIs.^49^ ^,^ ^165^ ^,^ ^166^ Thus, targeting the microbiota to enhance antitumor immunity is an emerging strategy.

#### 6.1.1 Innate immune response

**Figure 2.**
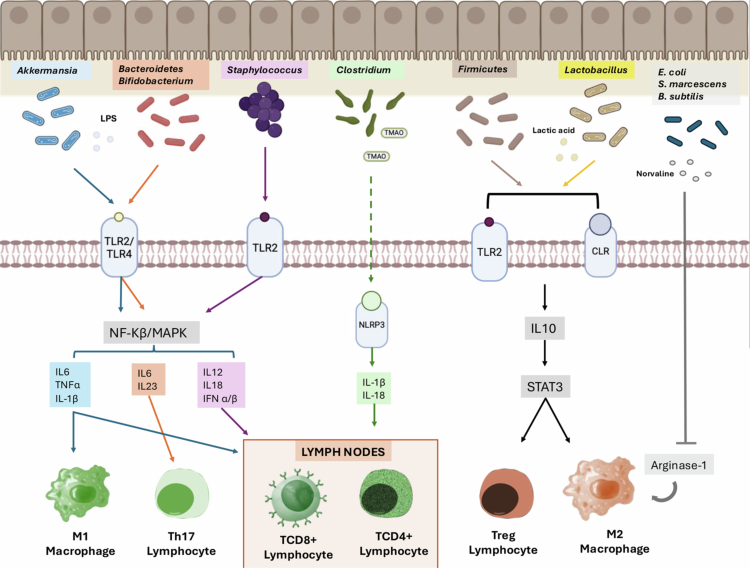
Modulation of the immune response mediated by the intestinal microbiome and microbial metabolites in TNBC. Bacterial components and microbiota-derived metabolites, including lipopolysaccharide (LPS), lactic acid, norvaline, and trimethylamine *N*-oxide (TMAO), are recognized at the intestinal epithelium by pattern-recognition receptors (PRRs), such as Toll-like receptors (TLR2 and TLR4), C-type lectin receptors (CLR). Engagement of these receptors activates proposed intracellular signaling cascades involving NF-κB and MAPK, leading to the production of cytokines (IL-6, TNF-*α*, IL-1β, IL-12, IL-18, and type I interferons). These signals promote macrophage M1 polarization, Th17 differentiation, and the activation of CD8⁺ and CD4⁺ T lymphocytes within lymph nodes, supporting antitumor immune responses in TNBC (green). However, other studies in tumor models have shown that Th17 lymphocytes exert context-dependent effects. In parallel, specific commensal bacteria, including *Lactobacillus* and members of the *Firmicutes* phylum, preferentially activate TLR2 and CLR signaling pathways, inducing the secretion of the anti-inflammatory cytokine IL-10. IL-10 signaling activates STAT3, which drives the transcription of genes associated with immune tolerance and tissue remodeling, promoting regulatory T cell (Treg) differentiation and polarization of macrophages toward an immunosuppressive M2 phenotype. Among these STAT3-dependent genes, arginase-1 plays a critical role by metabolizing L-arginine, thereby promoting M2 polarization. Although immune sensing is initiated in the gut, the resulting immunological signals exert systemic effects that shape the mammary tumor microenvironment, influencing the balance between pro-tumoral and antitumoral immunity in TNBC. Abbreviations: **LPS**: lipopolysaccharide; **TMAO**: trimethylamine *N*-oxide; ***E. coli***: *Escherichia coli*; *S. marcescens*: *Serratia marcescens*; *B. subtilis*: *Bacillus subtilis*; **TLR:** Toll-like receptor; **NLRP**: NOD-like receptor pyrin domain-containing protein; **CLR**: C-type lectin receptor; **NF-κB**: nuclear factor kappa-light-chain-enhancer of activated B cells; **MAPK**: mitogen-activated protein kinase; **IL:** interleukin; **TNF**: tumor necrosis factor; **IFN:** interferon; **STAT3**: signal transducer and activator of transcription 3; **Th:** T helper lymphocyte. [Created in Biorender: https://BioRender.com/dzuz3du]

The innate immune system provides the first barrier against microbes. Macrophages display M1 phenotypes that are stimulated mainly by interferon (IFN)-*γ*, LPS, lipoteichoic acid (LTA) or virus, eliciting a Th1-proinflammatory and antitumor response. Conversely, TAMs usually have a M2 phenotype, that are stimulated by interleukins, immune complex (IC) in combination with TLR ligands and promote a Th2-oriented and anti-inflammatory response that favors tumor progression.^49^ ^,^ ^165-167^ In a murine model of TNBC, *A. muciniphila* favors M1 polarization in TME via TLR2 and TLR4, activating NF-κB/MAPK proinflammatory signaling, as represented in Figure 2.^168^ ^,^ ^169^ Particularly, some multi-model studies have demonstrated that these bacteria exert their immunometabolic effects through specific outer membrane proteins such as Amuc_1100 that are resistant to pasteurization.^170^ Although *A. muciniphila* is generally considered a beneficial commensal, its biological effects appear to be highly context-dependent.^169^ LPS derived from *A.muciniphila* can be transported via extracellular vesicles and may promote TNF-*α*-mediated of cancer cell apoptosis within the tumor microenvironment, particularly when combined with inhibitors of apoptosis proteins (IAP) antagonists.^171^ In contrast, *Lactobacillus* supplementation promoted M2 polarization in the TME and tumor progression in TNBC mice, reversed by streptomycin.^100^ A similar effect was observed in the mammary microbiome, *Lactobacillus* activity has been linked to carcinogenesis-related gene expression (e.g., phospholipase A2, cyclin D1).^172^ *Lactobacillus*-derived ligands bind to TLR2 receptors in the intestinal environment which can favor tumor immunosuppression IL-10/STAT3 pathways which influence tumor immunity promoting the infiltration of M1 macrophages and CD8 T cells within the TME.^173^ These findings contrast with reports in other tumor types, where *Lactobacillus* species have been associated with enhanced antitumor immunity, underscoring the context-dependent effects.^174^ Shiao et al.^174^ detected in E0771 mammary tumors antibiotics that trigger fungal overgrowth (e.g., *Saccharomycetales*) increase M2 infiltration activating C-type lectin-like receptors (CLRs) (e.g. Dectin-1) in intestinal immune cells, whereas commensal gut taxa in untreated mice such as *Clostridiales*, *Burkholderiales*, and *Bifidobacterium* correlate with fewer M2 macrophages, thereby contributing to a systemic immune reprogramming capable of modulating the tumor microenvironment.^175^

N2 neutrophils also promote immunosuppression and pre-metastatic niches.^166^ Dutta et al.^70^ detected that dysbiosis in TNBC models increased N2 infiltration in intestine, circulation, and bone marrow, enhancing tumor cell extravasation and immune evasion. Although direct evidence in breast cancer models is limited, recent work in murine tumor models has shown that certain gut microbes can enhance antitumor immunity by promoting the maturation and migration of conventional dendritic cells (DC) from the gut to the tumor microenvironment, where they prime CD8⁺ T cells and improve response to immune checkpoint blockade.^176^

#### 6.1.2 Adaptive immune response

GALT plays a central role in lymphocyte priming, after which activated immune cells enter the peripheral circulation and migrate into the TME as TILs.^177^ In BC (including TNBC), several bacterial taxa, such as *Streptococcus*, *Propionibacterium*, *Staphylococcus* and *Acinetobacter*, have been correlated with increased lymphocyte recruitment within the TME. Beyond this gut–tumor immune axis, emerging evidence indicates that the intratumoral microbiome of TNBC, enriched in specific taxa such as *Fusobacterium* and *Citrobacter*, can directly modulate local immune responses, highlighting a complementary and mechanistically distinct layer of immune regulation compared with microbiota-driven systemic immune priming.^61^ ^,^ ^64^ ^,^ ^65^ However, the specific immune receptors and signaling pathways activated by these tumor-associated microorganisms remain to be clearly defined.

B cells have emerged as key modulators of the tumor immune microenvironment in TNBC. Recent studies indicate that T/B cell crosstalk critically shapes antitumor immunity. In several studies, mainly conducted in mouse models*, A. muciniphila* and microbiota-derived signals associated with *Bifidobacterium* and *Lactobacillus* have been shown to promote B-cell differentiation, IgA and IgG production, and antigen-specific T-cell responses, contributing to immune homeostasis at mucosal sites and generating systemic immune signals.^167^ ^,^ ^169^ ^,^ ^178^ Although direct evidence in TNBC remains limited, these systemic immunomodulatory effects suggest a potential role for gut microbiota–driven B-cell responses in shaping humoral immunity and T–B cell interactions within the TNBC tumor microenvironment.^179^

Numerous microbial signals within the intestinal lumen drive the differentiation of CD4⁺ T cells into effector subsets. Functionally, these immune cell subsets display distinct roles: Th17 cells enhance antibacterial responses and may contribute to cytotoxicity, while Tregs suppress immunity, particularly CD8⁺ T-cell–mediated responses.^165^ Consequently, maintaining the Th17/Treg balance has been shown to be crucial in TNBC. Fucoidan that promotes SCFA enrichment through the microbiota (e.g., *Bifidobacterium* and *Faecalibaculum*), promotes systemic immune modulation through microbiota-derived metabolites, leading to reduced Treg accumulation and reprogramming of the tumor microenvironment in TNBC.^180^ Conversely, in a murine model of mammary carcinoma, pharmacological inhibition of the Hedgehog pathway (e.g., vismodegib) was associated with a reduced *Firmicutes*/*Bacteroidetes* ratio, enhanced Th17 polarization, and CD8⁺ T-cell proliferation within the intestinal immune compartment thereby promoting systemic immune effects.^181^ Dysbiosis may therefore disrupt Th17/Treg homeostasis, favoring tumor growth.

Moreover, CD8⁺ T cells are critical effectors, eliminating tumor cells via FAS/FASL and serving as central mediators of anti-PD-1/PD-L1 immunotherapy.^165^ A previous multi-model study showed that elevated levels of choline derived from *Clostridium* species are associated with increased secretion of IL-18 and IL-1β in intestinal immune cells, potentially through activation of the NLRP3 inflammasome, thereby, promoting a systemic immune response that enhances CD8+ T-cell infiltration into tumors. In this context, the microbiota-derived metabolite TMAO, activates endoplasmic reticulum stress in tumor cells, leading to the activation of PKR-like ER kinase (PERK) and GSDME dependent pyroptosis. This results in the release of immunogenic damage-associated molecular patterns (DAMPs), activation of CD8⁺ T cells, and thereby enhancing the response to anti-PD-1 therapies.^98^ Conversely, this antitumor effect was inhibited by the TMAO-blocker 3-dimethyl-1-butanol (DMB).^98^ ^,^ ^182^ Among microbiota-derived metabolites, TMAO currently represents one of the clearest examples of a mechanistically defined immune-modulatory pathway specifically demonstrated in TNBC.

In a murine TNBC model, *Lactobacillus* supplementation increased lactate, which suppressed IFN-*γ*, and inhibited infiltration of CD4⁺ and CD8⁺ T lymphocytes within the tumor.^100^ In contrast, *S. aureus* and *S. hominis* enhanced CD8+ infiltration and inhibited tumor growth in murine and patient cohorts.^92^ Specific taxa of the tumor microbiome also modulate T cell infiltrates. Intratumoral bacterial taxa have been associated with distinct T cell-related immune signatures in BC. In particular, *Stenotrophomonas* and members of the *Conexibacteraceae* family have been positively correlated with enhanced CD8⁺ T cell activation and cytotoxic immune programs. In contrast, enrichment of *Staphylococcus* and *Streptococcus* within the breast tumor tissue microbiome has been associated with pro-inflammatory T cell response.^177^

Although the pathways described above are the best characterized, both gut and mammary microbiota can induce context-dependent immune responses. These effects are influenced by multiple factors, including the stage of tumor development, the local inflammatory state, and interactions among co-existing microbial communities. Moreover, immune cells modulated by microbial cues may exert either protumoral or antitumoral functions depending on these contextual variables. Nevertheless, these findings, summarized in Table 3 and represented in Figure 2, demonstrate how microbiota composition influences innate and adaptive immunity, suggesting that interventions targeting microbial composition and metabolites could enhance immunotherapy outcomes in TNBC.

**Table 3.** Mechanisms of Interaction Between Immune Cell Subtypes, Gut Microbiota, and Tumor Cells in Triple-Negative Breast Cancer (TNBC).

Cell type	Microbiome axis	Overall effect	Immune mechanism	Ref.
Macroph. M1 M2	Bacterial LPS	Antitumoral	1. Activate CD8+ T cells and promote Th1 polarization through cytokine production (IL-12, TNF-*α*, IL-1β and IFN-*γ*). 2. Facilitate the infiltration of NK cells and CD8⁺ T cells.	[168,183]
	*Akkermansia muciniphila*			
	*Lactobacillus*	Protumoral	1. Suppress immune responses (CD8⁺ T cells and NK cells) via IL-10 secretion and increased PD-L1 expression. 2. Recruit Tregs through TGF-*β* secretion. 3. Promote angiogenesis.	[100,174,183]
	*Saccharomycetales*			
Neutroph. N2	Dysbiosis	Protumoral	1. Suppress immune responses (CD8⁺ T cells and NK cells) via secretion of TGF-*β* and prostaglandin E. 2. Promote angiogenesis.	[70,166]
CD4 + Th17 Treg	Increased *Bacteroidetes* and decreased *Firmicutes*	Antitumoral	1. Activate CD8⁺ T cells and NK cells via secretion of IL-21 and IL-22. 2. Can transdifferentiate into Th1 lymphocytes.	[180,181]
	Decreased beneficial bacteria: *Lactobacillus* *Bifidobacterium* *Faecalibaculum*	Protumoural	1. Inhibition of DC affecting T-lymphocyte activation capacity. 2. Suppress immune responses (CD8⁺ T cells) through the secretion of IL-10, TGF-*β*, and IL-35. 3. Produce adenosine, which is toxic to T cells, and consume IL-2, depriving T cells of this critical growth factor	
CD8+	*Clostridium* (TMAO)	Antitumoral	1. Destroy tumor cells via the FAS/FASL pathway. 2. Activate other immune cells, such as M1 macrophages, NK cells, and Th1 lymphocytes, through the release of IFN-*γ* and TNF-α.	[92,98]
	*Staphylococcus:* *S. aureus* *S. hominis*			
	*Akkermansia*			

This table summarizes the microorganisms and microbial metabolites involved in the activation of the immune system, as well as the mechanisms by which they contribute to the positive or negative modulation of tumor cell progression in TNBC and their related references. Abbreviations: **Macroph**.: Macrophages; **LPS**: Lipopolysaccharides; **Th1**: T helper type 1; **IL**: Interleukin; **TNF**: Tumor Necrosis Factor; **IFN**: Interferon; **NK**: Natural killer; **PD-L1**: Programmed Death-Ligand 1; **TGF**: Transforming growth factor; **Neutroph**.: Neutrophil; **Treg**: Regulatory T cells; **DC**: Dendritic Cell; **TMAO**: Trimethylamine *N*-oxide.

## 7. Therapeutic strategies targeting the microbiota in TNBC

TNBC remains a challenging breast cancer subtype due to the lack of hormone receptors and HER2 amplification, which limits classical targeted therapies and increases reliance on chemotherapy and, more recently, immune checkpoint blockade in selected settings. Both modalities can reshape the gut microbiota and its metabolic output, with potential downstream effects on systemic immune tone and, secondarily, the tumor microenvironment (TME). Interest in microbiota modulation has therefore increased, as microbial communities and their metabolites can influence antitumor immunity, inflammatory tone, and treatment responsiveness.

### 7.1 Probiotics, prebiotics, and synbiotics

One of the most explored microbiota-targeting strategies involves probiotics (live microorganisms conferring health benefits when administered in adequate amounts) and prebiotics (nondigestible substrates that selectively promote beneficial taxa), including combined “synbiotic” formulations. Typically, these interventions are administered orally (supplements, food, capsules, etc.), although emerging approaches also include microbe-derived products, such as membrane vesicles (MVs) or metabolites. In preclinical TNBC models, several probiotic-related approaches have shown antitumor or immune-modulatory effects. For example, *Lactobacillus helveticus* strains modulated immune responses in 4T1 tumors,^184^ and probiotic-rich kefir water exerted anti-metastatic and anti-angiogenic effects in murine TNBC models.^185^ In addition, membrane vesicles from *Bifidobacterium* demonstrated direct pro-apoptotic activity and reduced TNBC growth,^186^ supporting the idea that “microbe-derived products” may retain functional activity.

Beyond intrinsic effects, probiotics may influence treatment response through drug-microbiome metabolic crosstalk and immune modulation. For example, bacterial metabolism enhanced lapachol cytotoxicity *in vitro*, ^187^ and probiotic supplementation with *E. coli* Nissle 1917, *L. johnsonii* and *A. muciniphila* improved antitumor immunity and ICI efficacy in TNBC mouse models.^188^ ^,^ ^189^ In parallel, oral prebiotics have been proposed as supportive strategies after standard therapy, with microbiome profiling explored in treated BC cohorts.^190^ Clinical translation of probiotic-based approaches is emerging. For example, a multicenter randomized phase II clinical trial (NCT06768931) is evaluating the safety and efficacy of an oral probiotic compound (Biolosion) in combination with neoadjuvant therapy in locally-advanced TNBC. Similarly, a recently initiated randomized, double-blind multicenter clinical trial (NCT07191405) is evaluating the addition of *L. johnsonii* supplementation to standard systemic therapy in multiple solid tumors, including TNBC cases, aiming to determine its impact on treatment outcomes and microbiome composition.

A clinically relevant aspect is the potential role of microbiota-targeted interventions in treatment tolerance. In a randomized placebo-controlled trial, probiotics reduced the incidence of chemotherapy-associated hand–foot syndrome and oral mucositis and improved gut microbial diversity in BC patients.^191^ Similarly, synbiotic supplementation improved gastrointestinal symptoms and reduced fatigue and nausea in women receiving chemotherapy.^192^ These supportive-care signals may be valuable even where antitumor efficacy remains unproven.

**Safety and limitations.** Despite promise, probiotic effects are highly dependent on strain, dose, and host context, and product heterogeneity/quality remains a major limitation. In immunocompromised settings, safety considerations are essential (e.g., severe mucositis, central lines, profound neutropenia). From a mechanistic perspective, genus-level assumptions should be avoided, as taxa can exert divergent effects depending on strain and ecological context (a theme consistent with bidirectional metabolite effects discussed in Section 5).

**Evidence level in TNBC:** predominantly preclinical with emerging clinical supportive-care data; antitumor efficacy in TNBC patients remains to be validated in standardized trials.

### 7.2 Dietary interventions

Diet is a major determinant of microbiota structure and metabolite production and can rapidly and reproducibly remodel the gut microbiome.^193^ Epidemiologic and preclinical literature suggests that dietary patterns associated with metabolic dysregulation may correlate with increased TNBC risk, whereas plant-forward dietary patterns may be protective.^194^ Mechanistically, diet-driven microbiome changes are relevant because they can alter microbial metabolite pools (e.g., SCFAs, bile acids, indole derivatives) and thereby modulate immune tone and tumor biology.

In murine models, high-fat diet promoted tumor initiation/progression and induced CD8⁺ T-cell exhaustion, impairing responses to anti-PD-1 therapy,^195^ directly supporting the concept that diet–microbiome interactions can shape ICIs sensitivity. In contrast, a high-fiber Mediterranean-style dietary pattern has been associated with reduced BC risk across meta-analytic evidence on dietary fiber.^196^ Currently, a randomized controlled clinical trial (NCT07311083 - BallastImmun) is looking to promote and implement a high-fiber diet among TNBC patients, and investigate its expected effect on the pCR. Mediterranean dietary patterns are also rich in polyphenols, which have been linked to reduced inflammatory biomarkers and may contribute to anticancer effects through anti-inflammatory and antioxidant mechanisms, potentially intersecting with microbiome-mediated immunomodulation.^197^

**Evidence level in TNBC:** strong biologic plausibility and preclinical support for immunotherapy-relevant effects; clinical evidence in TNBC is largely associative and requires prospective validation and intervention trials (ideally with microbiome/metabolome endpoints).

### 7.3 Antibiotics and risk of dysbiosis

A major translational concern is the widespread use of antibiotics, which can cause dysbiosis and potentially blunt antitumor immunity. Preclinical work shows reduced efficacy of ICIs in antibiotic-exposed models, consistent with microbiome-dependent immune priming.^179^ In clinical cohorts, antimicrobial exposure within months prior to systemic therapy has been associated with worse cancer outcomes,^198^ and antibiotics administered during neoadjuvant therapy have been linked to reduced efficacy in breast cancer cohorts that include TNBC patients.^199^ Recovery of gut microbiota after antibiotic exposure may take weeks, with healthy adult recovery estimated around 4–6 weeks in some settings,^200^ suggesting that timing relative to therapy could be critical.

Notably, TNBC-focused observational evidence has also supported a negative association between antimicrobial exposure and outcomes. Antibiotic exposure correlated with lower TIL infiltration and poorer survival in TNBC, with long-lasting associations reported in a large cohort.^201^ Additionally, intravenous antibiotic administration during chemotherapy was associated with inferior OS and PFS in early-stage TNBC.^202^

**Interpretation caveat.** These clinical observations may be influenced by confounding (e.g., “confounding by indication,” where infections and patient frailty drive both antibiotic use and outcomes). Nevertheless, the convergence of preclinical and clinical signals supports antibiotic stewardship and careful consideration of antibiotic timing in the context of chemoimmunotherapy whenever clinically feasible.

**Evidence level in TNBC:** consistent observational associations and supportive preclinical evidence; causal inference remains challenging and benefits from prospective trial embedding of microbiome endpoints.

### 7.4 Fecal microbiota transplantation

FMT restores microbial diversity by transferring a donor microbial community and has demonstrated high efficacy in recurrent *Clostridioides difficile* infection, reaching approximately 90% in landmark clinical experience.^203^ In oncology, FMT is at an earlier stage but is conceptually attractive as a way to overcome therapy-associated dysbiosis or to transfer responder-associated microbiome features.

In preclinical TNBC models, fecal transfer from ICI responders reduced tumor growth and improved immunotherapy response.^204^ Consistent with these findings, a phase II interventional clinical trial (NCT05286294 - MITRIC) is exploring FMT in patients resistant to ICIs using clinical responders as donors. Further supporting the translational potential of this approach, a recently initiated phase I/II trial (NCT07292142 - FRIDA) aims to assess the safety and feasibility of FMT in combination with neoadjuvant chemotherapy in TNBC patients. In addition, fecal transplants from post-bariatric surgery donors improved outcomes and increased ICIs efficacy in TNBC mouse models compared with pre-surgery donors,^205^ supporting the idea that metabolic state and microbial ecology jointly shape antitumor immunity.

**Safety and limitations.** FMT carries inherent risks because it transfers a complex ecosystem; safety concerns include transmission of pathogens and ecosystem disruption. Cases of multidrug-resistant *E. coli* bacteremia have been reported following FMT, underscoring the need for rigorous donor screening and standardized protocols.^206^ As a result, FMT implementation in TNBC requires careful risk–benefit assessment, strong regulatory oversight, and ideally movement toward more controlled alternatives (e.g., defined consortia) as the field matures.

**Evidence level in TNBC:** promising preclinical data; early clinical exploration is ongoing; no TNBC-specific data yet supports routine integration as an add-on to standard regimens outside trials.

### 7.5 Precision microbiome modulation and metabolite-centric (“postbiotic”) approaches

As the field shifts from descriptive “who is there” microbiome profiling toward functional “what they do,” metabolite-centric strategies become particularly relevant for TNBC immuno-oncology. Rather than broad, non-specific modulation, emerging approaches include: (i) targeting specific microbial pathways linked to immunotherapy response, (ii) leveraging engineered bacteria to deliver immunomodulatory payloads, and (iii) focusing on “postbiotic” or microbe-derived products (e.g., vesicles or metabolites) that may offer improved standardization.

A representative example is the use of engineered bacteria delivering PD-L1 nanoantibodies directly into the TME, which achieved marked tumor reduction; co-administration with *L. reuteri* reduced intestinal inflammation in the reported system.^207^ Conceptually, metabolite/pathway targeting could also include enzyme-level interventions within microbiome–host metabolic interfaces relevant to TNBC biology (e.g., *β*-glucuronidase within the estrobolome, discussed previously), with the goal of improving mechanistic specificity and translational control.^113^ These precision approaches align with the broader theme of this review: microbial metabolites as actionable intermediates connecting microbial ecology to immune remodeling.

**Evidence level in TNBC:** early-stage and largely preclinical/conceptual; strong rationale for development given the bidirectional and context-dependent effects of microbiota-derived metabolites discussed in Sections 5-6.

Collectively, microbiota-targeted strategies offer a plausible route to modulate systemic and intratumoral immunity in TNBC. However, translation requires standardized interventions, careful safety assessment, and prospective clinical validation incorporating microbiome–metabolome endpoints. The most promising path forward is likely a precision framework that prioritizes functional mechanisms (metabolites and pathways) over taxonomy alone and embeds microbiome modulation into trial designs aligned with TNBC chemoimmunotherapy regimens. Supplementary Table 2 summarizes microbiota-targeted strategies in TNBC, distinguishing supportive-care applications from approaches with potential antitumor relevance. To enhance the clinical relevance of these approaches, ongoing early-phase clinical trials (Phase I/II) evaluating microbiome-targeted or microbiome-modulating interventions in TNBC are summarized in Supplementary Table 3.

Delivery platforms and regulatory layers (nanocarriers, exosomes, microRNAs…) achieving such precision will likely depend not only on selecting the right microbial pathways, but also on how these interventions are delivered and regulated in vivo. Beyond diet, probiotics, and FMT, next-generation strategies may require precision delivery of metabolite-centric (“postbiotic”) interventions to maximize efficacy and safety. Nano-engineered carriers and exosome-based delivery systems can improve stability, tissue targeting, and controlled release of immunomodulatory metabolites or enzyme inhibitors, potentially enabling synergy with ICIs by promoting immunogenic cell death, improving antigen presentation, and limiting myeloid-driven suppression. In parallel, dysregulated microRNAs—particularly those intersecting the p53 stress-response axis—represent a plausible mechanistic layer linking metabolic rewiring, immune escape, and therapeutic resistance in TNBC. While these approaches remain emerging, integrating microbiome/metabolite biology with delivery technologies and regulatory circuits (e.g., microRNA networks) may provide a rational route to overcome TNBC heterogeneity and drug resistance.^208-210^

## 8. Conclusions

TNBC remains a paradigmatic immunogenic yet immunotherapy-refractory disease, where clinical benefit from ICIs is confined to subsets of patients and resistance—primary or acquired—remains frequent. The emerging literature reviewed here supports the concept that the gut and tumor-associated microbiota are not passive bystanders but active components of the TNBC ecosystem, capable of shaping immune tone, myeloid polarization, T-cell fitness, and key metabolic programs.^211^ Microbiota-derived metabolites—particularly SCFAs, indole–tryptophan derivatives, bile acids, polyamines, and other small molecules—represent actionable molecular intermediates linking microbial ecology to host immunity and tumor biology.^165^ Importantly, these metabolites can exert context-dependent effects that vary with dose, compartment, receptor expression, tumor subtype, and treatment context, underscoring that “beneficial” versus “detrimental” microbial signals cannot be universally assigned without mechanistic and clinical stratification (Table 2).

Despite accumulating associations, translation into clinical practice is currently limited by key knowledge gaps: scarce TNBC-specific mechanistic validation, heterogeneous sampling and analytic pipelines, and incomplete control of confounders (diet, medications, antibiotics, obesity, geography).^212^ Nevertheless, early studies—including emerging clinical datasets and our preliminary observations in neoadjuvant chemoimmunotherapy—support the feasibility of using integrated microbiome–metabolome signatures to refine patient stratification (e.g., pCR likelihood) and to nominate candidate pathways for therapeutic modulation. Collectively, the field is moving from descriptive “who is there” microbiome profiling toward functional “what they do” metabolite-centric biology, which is particularly relevant for immuno-oncology.

## 9. Future directions

Bridging the translational gap. A recurrent challenge in the microbiome–immunotherapy field is that robust preclinical effects do not always translate into consistent clinical benefit. In TNBC, several factors likely contribute: (i) strong inter-individual variability in baseline microbiome composition shaped by geography, diet, medications (antibiotics, PPIs, metformin/NSAIDs), and body mass index (BMI) or comorbidities; (ii) differences between murine SPF/gnotobiotic settings and human exposures; (iii) heterogeneity in treatment regimens, endpoints, and sampling timepoints (often cross-sectional rather than longitudinal); and (iv) technical batch effects from heterogeneous stool/tumor processing, sequencing pipelines, and metabolomics platforms. These issues can inflate apparent associations, obscure reproducible signals, and contribute to inconsistent clinical findings unless sampling and analytics are harmonized and confounders are systematically captured and adjusted.

1) *Build TNBC-specific causal evidence across model systems.*
  Priority should be given to mechanistic studies in TNBC-relevant platforms that can disentangle host–microbe–tumor interactions: immune-competent organoid co-cultures, syngeneic and humanized mouse models, gnotobiotic systems, and defined microbial consortia. These models should be used to validate whether candidate metabolites directly reprogram antitumor immunity (e.g., CD8⁺ stemness/exhaustion, DC priming, myeloid suppressive programs) and whether they synergize with chemo-ICI regimens used in TNBC.
2) *Move from relative abundance to functional attribution.*
  Future work should combine shotgun metagenomics (and, where possible, metatranscriptomics) with targeted, absolute metabolite quantification and pathway-level inference to link taxonomy to enzymatic capacity. Stable isotope tracing and spatial metabolomics/imaging mass spectrometry represent particularly powerful approaches to determine whether key metabolites originate from microbiota vs host/tumor compartments and to map metabolite exposure to immune-cell states within the TME.
3) *Standardize study design and metadata to enable cross-cohort comparability.*
  Multi-center, longitudinal studies with harmonized sample collection (stool, blood, and ideally paired tumor tissue), standardized storage/processing, and rigorous negative controls (especially for low-biomass tumor samples) are essential. Comprehensive metadata—including diet, BMI/adiposity, antibiotics, PPIs/metformin/NSAIDs, and treatment timing—should be systematically captured to reduce confounding and improve generalizability of microbial/metabolite biomarkers.
  Practical workflow recommendations:
  • Sampling/timepoints: pre-treatment baseline + early-on-treatment (e.g., 2–3 weeks) + pre-surgery/progression; collect stool and blood systematically, and paired tumor tissue when feasible.
  • Pre-analytics: uniform collection kits, cold-chain timing, aliquoting strategy, and storage conditions; record time-to-freeze and freeze–thaw cycles.
  • Controls: include extraction blanks, library blanks, and mock communities in every batch; for low-biomass tumor work, include tissue-adjacent negatives and reagent controls.
  • Assays: combine shotgun metagenomics (± metatranscriptomics where feasible) with targeted absolute quantification of prioritized metabolites (e.g., SCFAs, bile acids, indoles, estrogen-related metabolites) and immune phenotyping (flow cytometry/cytokines/TILs signatures).
  • Analysis plan: pre-register primary endpoints, perform batch correction, adjust for key confounders, report effect sizes, and validate predictive models with external cohorts when available.
4) *Develop clinically actionable biomarker panels rather than single markers.*
  Given the multidimensional nature of microbiome effects, the most robust predictors of response to ICIs are likely to be composite models integrating (i) metabolite panels (e.g., SCFAs, bile acid, and indole-derived signatures), (ii) microbial functional pathways/genes, and (iii) host immune features (TILs, myeloid states, cytokines, transcriptomic immune signatures, PD-L1) and clinical variables. Advanced computational approaches (network analysis, causal inference frameworks, and machine learning with external validation) should be used, but must be paired with mechanistic validation to avoid spurious correlations.
5) *Prioritize rational microbiome interventions and “postbiotic” strategies.*
  Interventional studies in TNBC should evolve beyond broad probiotics toward precision approaches, including diet/fiber/polyphenol interventions, engineered consortia, metabolite supplementation or delivery platforms, and targeted inhibition of deleterious pathways (e.g., specific enzyme targets within tryptophan metabolism or estrogen deconjugation). Beyond microbiome-directed interventions, strategies aimed at overcoming TNBC heterogeneity and resistance should also consider tumor-intrinsic regulatory mechanisms and advanced delivery technologies. Dysregulated microRNAs within the p53 signaling pathway have been implicated in breast cancer biology, with particular relevance to TNBC progression, therapeutic resistance, and potential biomarker development.^208^ Moreover, exosome-based delivery platforms can be engineered to transport therapeutic cargos, including siRNA, and have shown potential to suppress metastatic dissemination in breast cancer.^209^ Nanomedicine approaches designed to enhance immunogenic cell death may further remodel the tumor immune microenvironment and improve the efficacy of cancer immunotherapy.^210^ These complementary strategies could eventually be combined with microbiome- or metabolite-centered interventions to develop more effective multidimensional therapeutic approaches in TNBC. Importantly, safety and regulatory considerations are central—especially for FMT—necessitating standardized donor screening, strain-level characterization, and careful monitoring for infectious and metabolic adverse events.
6) *Clarify the estrobolome–immune interface in TNBC and ER-low disease.*
  Although TNBC is defined by ER/PR negativity, ER-low tumors and non-classical estrogen signaling (ERβ/GPER) may intersect with immune regulation and microbial estrogen metabolism. Future studies should explicitly stratify by receptor status (including ER-low), evaluate *β*-glucuronidase activity alongside systemic estrogen metabolites, and test whether estrogen-like microbial metabolites influence immune infiltration and ICI response in a subtype-aware manner.
7) *Embed microbiome endpoints into prospective TNBC immunotherapy trials.*
  The fastest route to translation will be prospective integration of microbiome/metabolome sampling into neoadjuvant and metastatic TNBC trials, enabling temporal mapping of microbiota dynamics during therapy and linking them to clinical endpoints (pCR, event-free survival, toxicity) and immune remodeling. Such designs will facilitate biomarker qualification and identify windows where intervention is most likely to improve outcomes.

## Closing perspective

Overall, the microbiota–metabolite axis represents a promising, mechanistically plausible layer of regulation of TNBC immunity and immunotherapy response. The next phase of the field should focus on TNBC-specific causality, standardized multi-omics, and biomarker-driven interventional trials to move from associative signatures to actionable precision strategies that improve outcomes for patients with this aggressive subtype.

## Supplementary Material

Supplementary Material New_Supplementary_tables_GutMicrobes_2.docx

## Data Availability

Not applicable.

## Abbreviations

2-OHE2: 2-hydroxyestradiol
γ-Glu-Trp: γ-glutamyltryptophan
γ-Glu-Glu: γ-glutamylglutamate
AA: Amino acid
AKT: AKT serine/threonine kinase
APCs: Antigen-presenting cells
Arg-1: Arginase 1
ATP: Adenosine triphosphate
AUC: Area under the curve
BC: Breast cancer
BFT: *Bacteroides fragilis toxin*
BL1: Basal-like 1
BLIA: Basal-like immunoactivated
BMI: Body mass index
BRCA1/2: Breast cancer gene 1/2
C1: Cluster 1
CagA: Cytotoxin-associated gene A
CAFs: Cancer-associated fibroblasts
CAR T cells: Chimeric antigen receptor T cells
CLRs: C-type lectin receptors
cDCs: Conventional dendritic cells
CSC: Cancer Stem Cell
CT: Chemotherapy
CTLA-4: Cytotoxic T-lymphocyte–associated protein 4
CDT: Cytolethal distending toxin
DCA: Deoxycholic acid
DC: Dendritic cell
DMB: 3,3-dimethyl-1-butanol
DNMT: DNA methyltransferase
E1: Estrone
E2: 17β-estradiol
E3: Estriol
EGFR: Epidermal growth factor receptor
EMT: Epithelial–mesenchymal transition
ERs: Estrogen receptors
FadA: Fusobacterium adhesin A
FAS: Fas cell surface death receptor
FASL: Fas ligand
FFPE: Formalin-fixed, paraffin-embedded
FMT: Fecal microbiota transplantation
GALT: Gut-associated lymphoid tissue
GEO: Gene expression omnibus
GPER: G protein–coupled estrogen receptor
HDACs: Histone deacetylases
HER2: Human epidermal growth factor receptor 2
HIF: Hypoxia-Inducible Factor
HR: Hazard ratio
IAA: Indole-3-acrylic acid
IAALD: Indole-3-acetaldehyde
IAPs: Inhibitors of apoptosis proteins
IC: Immune complex
ICIs: Immune checkpoint inhibitors
IDO: Indoleamine 2,3-dioxygenase
IFN: Interferon
IgA: Immunoglobulin A
iHDAC: Histone deacetylase inhibitor
ILs: Interleukins
iNOS: Inducible Nitric Oxide Synthase
IPA: Indole-3-propionic acid
IS: Indoxyl sulfate
LCA: Lithocholic acid
LPS: Lipopolysaccharide
LTA: Lipoteichoic acid
M1: M1-like macrophages
M2: M2-like macrophages
MAMPs: Microbe-associated molecular pattern
MAPK: Mitogen-activated protein kinase
MDA-MB: MD Anderson Cancer Center- Mammary Breast
mTOR: Mechanistic target of rapamycin
MPS2: Molecular phenotype subtype 2
MPs: Metalloproteinases
MVs: Membrane vesicles
MYC: MYC proto-oncogene
N2: N2-like neutrophils
NA: Nutriacholic acid
NACT: Neoadjuvant chemotherapy
NADH: Nicotinamide adenine dinucleotide
NaB: Sodium Butyrate
NF-κB: Nuclear factor kappa B
NK: Natural killer
NLRP3: NLR family pyrin domain containing 3
NLRs: NOD-like receptors
NRF2: Nuclear factor erythroid 2–related factor 2
NSAIDs: Nonsteroidal anti-inflammatory drugs
OS: Overall survival
OXPHOS: Oxidative phosphorylation
PARP: Poly(ADP-ribose) polymerase
pCR: Pathological complete response
PD-1: Programmed cell death protein 1
PD-L1: Programmed death-ligand 1
PERK: Protein kinase RNA-like ER kinase
PFS: Progression-free survival
PI3K: Phosphoinositide 3-kinase
pCR: Pathologic complete response
PPIs: Proton pump inhibitors
PRs: Progesterone receptors
PRRs: Pattern recognition receptors
RB1: RB transcriptional corepressor 1
RD: Residual disease
RFS: Recurrence-free survival
RNA-seq: Ribonucleic acid-sequencing
ROS: Reactive oxygen species
SAH: S-adenosylhomocysteine
SAHA: Suberoylanilide hydroxamic acid
SCFAs: Short-chain fatty acids
SPF: Specific Pathogen-Free
STAT3: Signal transducer and activator of transcription 3
TAMs: Tumor-associated macrophages
TC: Tumor cell
Tcf7: T-cell factor 7
TCGA: The Cancer Genome Atlas
TGF: Transforming growth factor
TILs: Tumor-infiltrating lymphocytes
TLRs: Toll-like receptors
TMAO: Trimethylamine N-oxide
TMB: Tumor mutational burden
TME: Tumor microenvironment
TNBC: Triple-negative breast cancer
TNF: Tumor necrosis factor
TP53: Tumor protein p53
Tregs: Tegulatory T cells
TSA: Trichostatin A
YY1: Ying Yang 1 transcription factor
VEGF: Vascular endothelial growth factor
