# Supplementary material for: Modulation of the response to immunotherapy in triple-negative breast cancer: the role of the microbiota and microbial metabolites in the tumor microenvironment
Source: Gut Microbes. 2026 Jul 8;18(1):2697600. doi: 10.1080/19490976.2026.2697600 (PMC13353786; doi:10.1080/19490976.2026.2697600)
Supplement: Supplementary Material — New_Supplementary_tables_GutMicrobes_2.docx [file KGMI_A_2697600_SM0029.docx]

**Supplementary Table 1. Formal molecular and metabolomic subtypes described in TNBC.** Abbreviations: **TNBC**. Triple Negative Breast Cancer; **pCR**: pathologic complete response; **CT**: chemotherapy; **IT**: immunotherapy; **MPS**: metabolic-pathway-based subtypes.

| Methodology | Study | Subtype | Characteristics | Ref |
| --- | --- | --- | --- | --- |
| Gene expression microarray. **Molecular subtypes.** | Lehmann et al (2011) | Basal-like 1 (BL1) | Highly proliferative tumors with strong replication stress. **Highest pCR rates to CT** | 11 |
|  |  | Basal-like 2 (BL2) | Growth factor–driven phenotype |  |
|  |  | Immunomodulatory (IM) | Strong immune-related gene expression signatures |  |
|  |  | Mesenchymal (M) | Invasive and migratory phenotype |  |
|  |  | Mesenchymal Stem-Like (MSL) | Stem-like and stromal-enriched profile |  |
|  |  | Luminal Androgen Receptor (LAR) | Luminal-like transcriptional program despite ER negativity |  |
| Gene expression and immune related signatures. **Molecular subtypes.** | Burstein et al. (2015) | Basal-like immunoactivated (BLIA) | Strong antitumor immune activation and inflammatory profile. **Best prognosis and response to CT+IT.** | 12 |
|  |  | Basal-like immunosuppressed (BLIS) | Immune-depleted and immunosuppressive phenotype |  |
|  |  | Mesenchymal (MES) | Mesenchymal and invasive tumor phenotype |  |
|  |  | Luminal Androgen Receptor (LAR) | Hormone-driven transcriptional program |  |
| Gene expression microarray. **Molecular subtypes**. | Lehmann et al. (refined, 2016) | Basal-like 1 (BL1) | Highly proliferative tumors with strong replication stress. **Highest pCR rates to CT** | 13 |
|  |  | Basal-like 2 (BL2) | Growth factor-dependent tumors |  |
|  |  | Mesenchymal (M) | Invasive and migratory phenotype. Influence of non-tumor component. |  |
|  |  | Luminal Androgen Receptor (LAR) | Luminal-like transcriptional program despite ER negativity |  |
| Gene expression and pathway analysis. **Molecular subtypes.** | FUSCC (Jiang et al. 2019) | Immunomodulatory (IM) | Immune-enriched tumors with active antitumor immune responses. **Best prognosis and response to CT+IT.** | 14 |
|  |  | Luminal Androgen Receptor (LAR) | Hormone-related transcriptional program despite ER negativity |  |
|  |  | Mesenchymal-like (MES) | Invasive and stromal-associated phenotype |  |
|  |  | Basal-like-immunosuppressed (BLIS) | Highly proliferative tumors with limited immune infiltration |  |
| Transcriptomics with metabolic gene signature. **Metabolomic subtypes.** | MPS (Gong et al. 2021) | MPS1 | Metabolically active but poorly proliferative tumors. Lipid metabolism, fatty acid synthesis and oxidation | 15 |
|  |  | MPS2 | Highly proliferative tumors with **favorable prognosis.** Glycolysis, nucleotide biosynthesis, oxidative phosphorylation |  |
|  |  | MPS3 | Aggressive tumors with poor outcomes. Mixed metabolic program with enhanced amino acid metabolism and stress-related pathways. |  |
| Liquid chromatography–mass spectrometry (LC–MS). **Metabolomic subtypes** | C classification (Xiao et al. 2022) | C1 | Metabolically active tumors with **favorable outcomes**. Enhanced lipid metabolism, mitochondrial activity, efficient energy utilization. | 16 |
|  |  | C2 | Intermediate phenotype. Mixed lipid and glycolytic metabolic programs. |  |
|  |  | C3 | Metabolically dysregulated and aggressive tumors. Glycolysis, amino acid metabolism, redox imbalance |  |

**Supplementary Table 2. Microbiome-modulating interventions in Triple-Negative Breast Cancer (TNBC): Clinical Intent, Timing and Level of Evidence.** Abbreviations: **NACT**: Neoadjuvant Chemotherapy; **TNBC**: Triple-Negative Breast Cancer; **ND**: Non-Determined; **ICI**: Immune Inhibitor Checkpoint; **FMT**: Fecal Microbiota Transplant.

| **Intervention** | **Study Type** | **Plausible Clinical Endpoint** | **Optimal Timing** | **Risks and Limitations** | **Level of Evidence in TNBC** | **Sample (n)** | **Ref** |
| --- | --- | --- | --- | --- | --- | --- | --- |
| **Interventions with Supportive Care Intent** | | | | | | | |
| Probiotics and synbiotics | Translational from Phase II Clinical Trial Cohorts (ChiCTR1900024927; IRCT20091114002709N56) | Reduction of treatment-associated toxicity; improved quality of life | During NACT | Strong strain and context-dependence; limited clinical validation and standardization | Limited TNBC clinical studies | Breast cancer patients with stage I-III (n=159)  Breast cancer patients with chemotherapy (n=67) | 190, 191 |
| Oral Prebiotics | Translational (TNBC patients) | Reduction of disease recurrence risk | After standard treatment | Very limited clinical data | Limited TNBC clinical studies | Breast cancer patients for 12 weeks for after surgery, chemotherapy, and radiation treatment (n=60) | 189 |
| Controlled antibiotic stewardship (precautionary) | Translational from Early Phase I ERA 2012 Clinical trial cohort (NCT01633762) | Prevention of iatrogenic dysbiosis that may worsen treatment response | Whenever clinically feasible | Confounding by indication; risk of undertreating infections | Consistent observational data; strong preclinical evidence | TNBC patients with stage I-III (n=772) | 200 |
| Postbiotics (microbe-derived products) | Translational (TNBC patients) | Improved standardization for newer approaches, mechanistic specificity and translational control | ND | Very limited clinical data | Predominantly preclinical | ND | 113 |
| **Interventions with Potential Antitumor Efficacy** | | | | | | | |
| Selected probiotics (*A. muciniphilia, E. coli Nissle*…) | Preclinical (murine models and cell cultures) | Enhancement of antitumor immunity; reduction of tumor growth; improved ICI response | During ICI therapy | Strong strain and context-dependence.; limited clinical validation and standardization | Predominantly preclinical | Murine cell line 4T1 (n=5mice/group) | 183- 187 |
| Dietary interventions (high-fiber/ Mediterranean) | Preclinical, meta-analysis and translational | Prevention of CD8+ T cell exhaustion and response to anti-PD-1 therapy | Pre-ICI and during treatment | No interventional TNBC trials; indirect effects | Preclinical, largely associative | Breast cancer murine model MMTV-PyMT and Breast cancer patients (n=78) | 194-196 |
| Controlled antibiotic stewardship (precautionary) | Translational (TNBC patients) and from Phase III Clinical Trial SUCCESS A (NCT 02181101) and SUCCESS C (NCT 00847444) cohorts | Preservation of ICI and NACT efficacy | Whenever clinically feasible | Confounding by indication; risk of undertreating infections | Consistent observational data; strong preclinical evidence | TNBC patients with stage I-III (n=772) and TNBC patients with primary surgery, adjuvant chemotherapy, and radiotherapy (n=1583) | 201,202 |
| Fecal Microbiota Transplant (FMT) | Translational (TNBC patients) | Improved ICI response; reduction of tumor growth | After failure of ICI-based therapy | Risk of pathogen transmission; logistical complexity | Pre-clinical and early-phase ongoing clinical trials | C57BL/6J breast cancer mice FMT controls or experimental groups (n = 10 mice/group) and fecal donor breast cancer patients (n=2) | 205 |
| Engineered bacteria/precision metabolic targeting | Preclinical (murine model) | Tumor reduction; enhanced therapy response | ND | Early-stage development, largely conceptual | Preclinical | Murine cell line 4T1 (n = 6 mice/group) | 207 |

**Supplementary Table 3**. **Ongoing early-phase clinical trials (Phase I/II) evaluating microbiome-targeted or microbiome-modulating interventions in TNBC.** Abbreviations: **FMT**: Fecal Microbiota Transplant; **NAT**: Neoadjuvant Therapy; **TNBC**: Triple-Negative Breast Cancer; **pCR**: Pathologic Complete Response; **FMD**: Fasting-mimicking diet; **N/A**: Not Applicable. *Not classified under conventional drug development phases.

| **Trial Identifier** | **Phase** | **Study Type** | **TNBC stage** | **Intervention Type** | **Effect on Microbiota** | **Primary endpoint** | **Status** |
| --- | --- | --- | --- | --- | --- | --- | --- |
| NCT05967533 | I | Single-arm | III-IV solid tumors (including TNBC) | Nutritional supplementation (Fermented wheat germ) | Direct (Microbiome- targeted) | Assess immunologic response of the nutritional supplementation in combination with immunotherapy | Recruiting |
| NCT07292142 (FRIDA) | I/II | Open label, non- randomized | No- metastatic (NAT indication) | FMT | Direct (Microbiome- targeted) | Safety and feasibility of FMT treatment in combination with NAT | Not yet Recruiting |
| NCT06768931 | II | Randomized, multicenter, controlled | II-III | Oral Probiotic (Biolosion) | Direct (Microbiome- targeted) | Efficacy and safety of Biolosion in combination with standard NAT | Recruiting |
| NCT05763992 (BREAKFAST-2) | II | Randomized, multicenter, open-label, two-arm, comparative | II-III | Fasting-mimicking diet (FMD) | Indirect (Microbiome- modulating) | Increase pCR, NAT efficacy | Recruiting |
| NCT07378306 (FACT-TN) | II | Open label, single arm | I-III | FMD | Indirect (Microbiome- modulating) | Efficacy and safety of a FMD intervention combined with standard NAT | Recruiting |
| NCT06831955 (LESLIE) | II | Randomized, multicenter, controlled | II-III | FMD + exercise | Indirect (Microbiome- modulating) | Treatment efficacy, tolerability, and quality of life | Recruiting |
| NCT07191405 | N/A* | Randomized, multicenter, controlled, double-blind | III-IV solid tumors (including TNBC) | Oral Probiotic (*Lactobacillus johnsonii*) | Direct (Microbiome- targeted) | Treatment efficacy and drug-related adverse reactions. | Not yet Recruiting |
| NCT07311083 (BallastImmun) | N/A* | Randomized, controlled, open-label | I-III | Diet (Switching to a high fiber diet) | Indirect (Microbiome- modulating) | Feasibility of the online integrative oncology group training program for the implementation of a high-fiber diet | Not yet Recruiting |
